# Albendazole reduces hepatic inflammation and endoplasmic reticulum-stress in a mouse model of chronic *Echinococcus multilocularis* infection

**DOI:** 10.1371/journal.pntd.0009192

**Published:** 2022-01-14

**Authors:** Michael Weingartner, Simon Stücheli, Fadi Jebbawi, Bruno Gottstein, Guido Beldi, Britta Lundström-Stadelmann, Junhua Wang, Alex Odermatt

**Affiliations:** 1 Division of Molecular and Systems Toxicology, Department of Pharmaceutical Sciences, University of Basel, Basel, Switzerland; 2 Institute for Infectious Diseases, Faculty of Medicine, University of Bern, Bern, Switzerland; 3 Institute of Parasitology, Department of Infectious Diseases and Pathobiology, Vetsuisse Faculty, University of Bern, Bern, Switzerland; 4 Department of Visceral Surgery and Medicine, University Hospital of Bern, Bern, Switzerland; University of Würzburg, GERMANY

## Abstract

**Background:**

*Echinococcus multilocularis* causes alveolar echinococcosis (AE), a rising zoonotic disease in the northern hemisphere. Treatment of this fatal disease is limited to chemotherapy using benzimidazoles and surgical intervention, with frequent disease recurrence in cases without radical surgery. Elucidating the molecular mechanisms underlying *E*. *multilocularis* infections and host-parasite interactions ultimately aids developing novel therapeutic options. This study explored an involvement of unfolded protein response (UPR) and endoplasmic reticulum-stress (ERS) during *E*. *multilocularis* infection in mice.

**Methods:**

*E*. *multilocularis-* and mock-infected C57BL/6 mice were subdivided into vehicle, albendazole (ABZ) and anti-programmed death ligand 1 (αPD-L1) treated groups. To mimic a chronic infection, treatments of mice started six weeks post *i*.*p*. infection and continued for another eight weeks. Liver tissue was then collected to examine inflammatory cytokines and the expression of UPR- and ERS-related genes.

**Results:**

*E*. *multilocularis* infection led to an upregulation of UPR- and ERS-related proteins in the liver, including ATF6, CHOP, GRP78, ERp72, H6PD and calreticulin, whilst PERK and its target eIF2α were not affected, and IRE1α and ATF4 were downregulated. ABZ treatment in *E*. *multilocularis* infected mice reversed, or at least tended to reverse, these protein expression changes to levels seen in mock-infected mice. Furthermore, ABZ treatment reversed the elevated levels of interleukin (IL)-1β, IL-6, tumor necrosis factor (TNF)-α and interferon (IFN)-γ in the liver of infected mice. Similar to ABZ, αPD-L1 immune-treatment tended to reverse the increased CHOP and decreased ATF4 and IRE1α expression levels.

**Conclusions and significance:**

AE caused chronic inflammation, UPR activation and ERS in mice. The *E*. *multilocularis*-induced inflammation and consecutive ERS was ameliorated by ABZ and αPD-L1 treatment, indicating their effectiveness to inhibit parasite proliferation and downregulate its activity status. Neither ABZ nor αPD-L1 themselves affected UPR in control mice. Further research is needed to elucidate the link between inflammation, UPR and ERS, and if these pathways offer potential for improved therapies of patients with AE.

## Author summary

Alveolar echinococcosis (AE) is a zoonotic disease, characterized by chronic progressive hepatic damage caused by the continuous tumor-like proliferation of the larval stage (metacestode) of the fox tapeworm *Echinococcus multilocularis*. Treatment of this fatal disease is limited to surgical intervention, preferably radical curative surgery if possible, and the use of parasitostatic benzimidazoles. It is not yet fully understood how the parasite can remain in the host’s tissue for prolonged periods, complicating the development of therapeutic applications. This work investigated an involvement of the unfolded protein response (UPR) and endoplasmic reticulum-stress (ERS) during *E*. *multilocularis* infection and upon treatment with either albendazole (ABZ) or anti-programmed death ligand-1 (αPD-L1) in mice. The results revealed increased expression levels of the ERS sensor ATF6 and of downstream target genes in liver tissue of *E*. *multilocularis-* compared to mock-infected mice. Additionally, hexose-6-phosphate dehydrogenase (H6PD), generating NADPH within the endoplasmic reticulum, and the lectin-chaperone calreticulin were increased in *E*. *multilocularis* infected liver tissue while the expression of the ERS associated genes ATF4 and IRE1α were decreased. The observed gene expression changes were at least partially reversed by ABZ treatment, which also reduced the AE-induced increase of the inflammatory cytokines IL-1β, IL-6, TNF-α and IFN-γ. PD-L1 blockade reversed the AE-induced changes of UPR and ERS associated proteins CHOP, ATF4 and IRE1α. Further investigation is needed to elucidate the link between inflammation and ERS in human patients with AE and whether modulation of these pathways may lead to improved therapy.

## Introduction

Alveolar echinococcosis (AE) is a severe helminth disease caused by accidental ingestion of eggs from the fox tapeworm *Echinococcus multilocularis* [1,2]. After an incubation period of 5 up to 15 years without perceivable symptoms, AE has a fatal outcome in up to 90% of cases when left untreated [3–5]. AE is characterized by a slow but progressive tumor-like growth of metacestodes (larval stage) mainly in the liver, with a tendency to spread to various organs like spleen, brain, heart and other tissues such as bile ducts and blood vessels [6–8]. The variable clinical outcomes of AE development depend on the immunological status, and the specific immunological profile with T cell exhaustion seems to play an important role in the established tolerance state in chronic AE [9–11].

Treatment by radical surgical resection is limited by the diffuse infiltrations of AE lesions in liver and other tissues in advanced cases [12,13]. If lesions cannot be completely removed by surgery, a lifelong medication is required, usually using benzimidazoles, which can cause adverse side effects. For example, several cases with hepatotoxic effects due to treatment with the benzimidazole albendazole (ABZ) were reported with various outcomes [14–17]. An inadequate adherence to chemotherapy, due to adverse side effects, and development of resistance can explain the relapsing spread of AE and a worsening general condition of patients with severe *E*. *multilocularis* infiltrations [11,18,19]. Recent experiments using mice indicated a requirement of functional T cell immunity for efficient treatment of AE with ABZ [20]. Considering these circumstances, the rising number of reported cases of AE especially in Europe and the lack of a curative drug treatment, emphasizes the necessity to further investigate the mechanisms underlying this threat and search for improved therapeutic options [21–26].

Several bacteria and viruses have been described to modulate unfolded protein response (UPR) and endoplasmic reticulum stress (ERS), either by bacterial virulence factors such as toxins (*e*.*g*. cholera toxin, pore-forming toxins) or by the increased demand of newly synthesized proteins for the production of virions [27–32]. Activation of the UPR via an induction of glucose-regulated protein 78 (GRP78) has previously been shown in cells infected with *Human immunodeficiency virus* (HIV) [31,33], *Dengue virus* (DENV) [34], *West Nile virus* (WNV) [35] or *Human cytomegalovirus* (HCMV) [36]. Moreover, facilitated replication of viruses and immune evasion represent key features following UPR activation by *Mouse hepatitis virus* (MHV) [37] and *Herpes simplex virus 1* (HSV-1) [38]. On the other hand, an ERS-induced upregulation of UPR-related genes was linked with an enhanced production of pro-inflammatory cytokines in monocytes and B-cells [39–41]. A modulation of the UPR pathway was reported not only during viral but also bacterial infections. *Legionella pneumophila* infection led to an inhibition of X-box binding protein 1 (XBP1) splicing in mammalian host cells, thereby suppressing the host UPR pathway [42]. *Mycobacterium tuberculosis* was found to induce ERS, indicated by increased CCAAT/enhancer-binding protein homologous protein (CHOP) and GRP78 protein levels in infected macrophages, leading to host cell apoptosis. Decreased levels of phosphorylated eukaryotic initiation factor 2α (eIF2α) in infected cells were associated with enhanced bacterial survival [43].

However, to date the knowledge of pathogen-induced ERS and UPR activation is incomplete; it is mainly limited to bacterial and viral infections and little is known on extracellular pathogens. A modulation of the host’s UPR with an upregulation of CHOP was observed in *Toxoplasma gondii* infected cells, leading to apoptosis of host cells [44]. Another study in a mouse model provided evidence that *Plasmodium berghei* exploits the host’s UPR machinery for its survival [45]. However, the involvement of ERS and UPR activation in *E*. *multilocularis* infection has not been studied in detail to our knowledge.

Several studies revealed a functional interaction between UPR/ERS signaling and the expression of microRNAs (miRs), small non-coding single stranded RNAs (17–24 nucleotides) that regulate the post-transcriptional levels of mRNAs by inhibiting their translation to proteins [46,47]. Silencing of miRs was found to be involved in ERS signaling and miRs act as effectors and modulators of the UPR and ERS pathways [48]. The miRs, isolated from human specimen, including urine, saliva, serum and tissues, are considered as biomarkers of several immune pathologies such as cancer, autoimmune diseases and viral or bacterial infections [49–55]. A recent study revealed miR-125b-5p to be elevated in the plasma of AE patients [56]. Furthermore, recent investigations provided evidence for a role of some miRs in the regulation of UPR signaling, with miR-181a-5p and miR-199a-5p shown to suppress the UPR master regulator GRP78 [48,57,58]. On the other side, UPR pathways also can affect the expression of some miRs, as shown by inositol-requiring enzyme 1α (IRE1α) that cleaves anti-apoptotic miR-17, miR-34a, miR-96 and miR-125b, preventing them from negatively regulating the expression of caspase 2 and thioredoxin-interacting protein [59,60]. In addition, the activation of protein kinase R (PKR)-like ER kinase (PERK) induces the expression of miR-30c-2-3p, which downregulates XBP1, representing a possible negative crosstalk between PERK and IRE1α [61]. Boubaker *et al*. [62] recently described a murine miR signature in response to early stage, primary *E*. *multilocularis* egg infection where the expression of several miRs was either decreased or increased in AE-infected compared to mock-infected mice.

The present study addressed how the expression of UPR- and ERS-related genes was affected in liver tissue in a mouse model of chronic *E*. *multilocularis* infection and whether alterations in miRs might be involved. Moreover, the effect of ABZ and αPD-L1 treatment on UPR and ERS pathways as well as on levels of proinflammatory cytokines in the liver were assessed. A better understanding of a contribution of proteins of the UPR and ERS pathways in the context of infectious diseases is of interest regarding the development of improved therapeutic strategies to cope with parasitic infections [63–65].

## Materials and methods

### Ethics statement

Animals were housed according to the Federation of European Laboratory Animal Science Association (FELASA) guidelines. The animal studies were performed in compliance with the recommendations of the Swiss Guidelines for the Care and Use of Laboratory Animals. The protocol used for this work was approved by the governmental Commission for Animal Experimentation of the Canton of Bern (approval no. BE112/17).

### Chemicals and reagents

Polyvinylidene difluoride (PVDF) membranes (Cat# IPVH00010, pore size: 0.45 μm), Immobilon Western Chemiluminescence horseradish-peroxidase (HRP) substrate kit, radioimmunoprecipitation assay (RIPA) buffer, β-mercaptoethanol, HRP-conjugated goat anti-mouse secondary antibody (Cat# A0168, RRID:AB_257867), rabbit polyclonal anti-hexose-6-phosphate dehydrogenase (H6PD) antibody (Cat# HPA004824, RRID:AB_1079037), protease inhibitor cocktail, dNTPs, and KAPA SYBR FAST qPCR kit (Cat# KK4618) were purchased from Merck (Darmstadt, Germany). RNeasy Mini kit and QIAcube were obtained from Qiagen (Venlo, Netherlands), GoScript reverse transcriptase (Cat# A5003) from Promega (Fitchburg, WI, USA), rabbit monoclonal anti-Lamin B1 antibody (Cat# ab133741, RRID:AB_2616597) and rabbit polyclonal anti-phospho (S724) IRE1α antibody (Cat# ab48187, RRID:AB_873899) from Abcam (Cambridge, UK) and mouse monoclonal anti-GRP78 antibody (Cat# 610978, RRID:AB_398291) from BD Bioscience (Franklin Lakes, NJ, USA). HRP-conjugated goat anti-rabbit secondary antibody (Cat# 7074, RRID:AB_2099233), mouse monoclonal anti-CHOP antibody (Cat# 2895, RRID:AB_2089254), rabbit polyclonal anti-calreticulin (CRT) antibody (Cat# 2891, RRID:AB_2275208), rabbit polyclonal anti-eIF2α antibody (Cat# 9722, RRID:AB_2230924), rabbit monoclonal anti-ATF4 antibody (Cat# 11815, RRID:AB_2616025), rabbit monoclonal anti-ATF6 antibody (Cat# 65880, RRID:AB_2799696), rabbit monoclonal anti-phospho (S51) eIF2α antibody (Cat# 3597, RRID:AB_390740), and rabbit monoclonal anti-XBP1-s antibody (Cat# 12782S, RRID:AB_2687943) were purchased from Cell Signaling (Cambridge, UK). Mouse monoclonal anti-PERK antibody (Cat# sc-377400, RRID:AB_2762850), anti-IRE1α antibody (Cat# sc-390960, RRID: N/A) and anti-ERp72 antibody (Cat# sc-390530, RRID: N/A) were obtained from Santa Cruz Biotechnology (Dallas, TX, USA). Pierce bicinchoninic acid protein assay kit, Nanodrop One C (Cat# 13-400-519), and Trizol total RNA isolation reagent and rabbit monoclonal anti-phospho (T980) PERK antibody (Cat# MA5-15033, RRID:AB_10980432) were purchased from Thermo Fisher Scientific (Waltham, MA, USA). Precellys-24 tissue homogenizer was purchased from Bertin Instruments (Montigny-le-Bretonneux, France). Primers for real-time quantitative polymerase chain reaction (RT-qPCR) were obtained from Microsynth (Balgach, Switzerland). TaqMan microRNA Assays, snoRNA234, TaqMan microRNA reverse transcription kit (Cat# 4366596), TaqMan fast advanced master mix (Cat# 4444556), TaqMan probes (Cat# 4427975, Assay IDs 000468, 000389, 000398, 000470, 121135_mat, 000416, 000417, and 001234) and ViiA 7 real-time PCR system (Cat# 4453545) were purchased from Applied Biosystems (Foster City, CA, USA). The mouse luminex cytokine kits and the BioPlex-200 platform were purchased from BioRad Labrotories, Cressier, Switzerland. Rat monoclonal anti-PD-L1 antibody (αPD-L1, Cat# BE0101, RRID:AB_10949073) was purchased from BioXCell (Lebanon, NH, USA). Rabbit polyclonal anti-calnexin (CNX) antibody (Cat# SAB4503258, RRID:AB_10746486) and all other reagents were purchased from Sigma-Aldrich (St. Louis, MO, USA).

### Animal experimentation and sampling

Animal experimentation, liver tissue extraction and corresponding liver tissue samples were previously described [10]. Briefly, female 8-week-old wild type C57BL/6 mice were randomly distributed into 6 groups with 6 animals per group: 1) mock-infected (corn oil treated) control mice (referred to as “CTRL”); 2) *E*. *multilocularis* infected, vehicle treated mice (referred to as “AE”); 3) *E*. *multilocularis* infected, ABZ-treated mice (referred to as “AE-ABZ”); 4) mock-infected, ABZ-treated mice (referred to as “ABZ”); 5) *E*. *multilocularis* infected, αPD-L1 treated mice (referred to as “AE-αPD-L1”); and 6) mock-infected, αPD-L1-treated mice (referred to as “αPD-L1”) (S1 Fig). All animals were housed under standard conditions in a conventional daylight/night cycle room with access to feed and water *ad libitum* and in accordance with the Federation of European Laboratory Animal Science Association (FELASA) guidelines. During the experimental period animals were examined weekly for subjective presence of health status and changes in weight. At the end of the experiment the mice were euthanized by CO_2_ and liver tissue was resected followed by immediate freezing in liquid nitrogen and storage at -80°C until use. Parasitic structures were visible only in the liver of some of the infected mice, and the upper part from the left lobe of the liver (1.5 × 1.5 cm) was collected, irrespective of the presence or absence of parasite lesions.

### Parasite preparation and secondary infection of mice by intraperitoneal administration

Infection with *E*. *multilocularis* by *i*.*p*. injection was conducted as previously described [10]. Briefly, *E*. *multilocularis* (isolate H95) was extracted and maintained by serial passages in C57BL/6-mice. Aseptic removal of infectious material from the abdominal cavity of infected animals was used for continuation of AE in mice. Collected tissue was grinded through a sterile 50 μm sieve, roughly 100 vesicular cysts were suspended in 100 μL sterile PBS and administrated *via* intraperitoneal injection to group 2 (“AE”), 3 (“AE-ABZ”) and 5 (“AE-αPD-L1”). Mice of the mock-infected groups 1 (“CTRL”), 4 (“ABZ”) and 6 (“αPD-L1”) received 100 μL of sterile PBS.

### Treatment

As described earlier [10], treatment of mice started 6 weeks after initial infection and continued for another 8 weeks (S1 Fig). Mice of the groups 1 and 2 (“CTRL”, “AE”, respectively) received 100 μL PBS by *i*.*p*. injection twice/week and 100 μL corn oil orally 5 times/week. Mice of group 3 and 4 (“AE-ABZ” and “ABZ”, respectively) received 100 μL corn oil containing ABZ (200 mg/kg body weight) orally five times/week and 100 μL PBS by *i*.*p*. injection twice/week. Mice of group 5 and 6 (“AE-αPD-L1” and “αPD-L1”, respectively) received αPD-L1 antibody in 100 μL PBS *via i*.*p*. injection twice/week (200 μg/injection) and 100 μL corn oil orally 5 times/week. At end of treatment all mice were euthanized.

### Luminex for quantification of hepatic cytokine levels

Cytokine levels in mouse liver samples were assessed undiluted using microsphere-based multiplex assays according to the manufacturer’s instructions; concentrations of the following cytokines were measured: IL-1β, IL-6, TNF-α and INF-γ, using mouse luminex cytokine kits (BioRad Labrotories, Cressier, Switzerland). At least 50 beads per analyte were measured on a Bioplex-200 platform (BioRad). Calibration was performed using BioPlex Manager version 4.1.1 by linear regression analysis using the four lowest standards provided by the manufacturer. If measured cytokine concentrations were below the detection limit, a value corresponding to the detection limit of the assay was used for statistical analysis.

### Analysis of protein expression by Western blotting

The procedures for liver sample preparation and Western blotting have been previously described [66]. Briefly, liver samples (approximately 7 mg) were homogenized (30s, 6500 rpm, at 4°C, using a Precellys-24 tissue homogenizer) in 450 μL RIPA buffer (50 mM Tris-HCl, pH 8.0, with 150 mM NaCl, 1.0% NP-40, 0.5% sodium deoxycholate and 0.1% sodium dodecyl sulfate) containing protease inhibitor cocktail and centrifuged (4 min, 16,000 × g, 4°C). Protein concentration was measured by a standard bicinchoninic acid assay (Pierce BCA Protein Assay Kit). Samples were boiled (5 min at 95°C) in Laemmli solubilization buffer (60 mM Tris-HCl, 10% glycerol, 0.01% bromophenol blue, 2% sodium dodecyl sulfate, pH 6.8, 5% β-mercaptoethanol). The protein extract (20 μg) was separated by 7.5–14% SDS-PAGE and blotted on PVDF membranes. The membranes were blocked (1 h, room temperature) in TBST-BSA, (20 mM Tris buffered saline with 0.1% Tween-20, 1% bovine serum albumin). All primary and secondary antibody dilutions and incubations were performed in TBST-BSA. For the detection of primary antibodies raised in rabbit, secondary HRP-conjugated goat anti-rabbit antibody was used. Primary antibodies raised in mouse were detected by HRP-conjugated goat anti-mouse antibody. Primary antibodies were incubated at 4°C over-night. Secondary antibodies were applied at room temperature for 1 h. Protein content was visualized by Immobilon Western Chemiluminescence HRP substrate. Protein bands were quantified by densitometry normalized to Lamin B1 protein levels using ImageJ software (version 1.53n). The applications of primary and secondary antibodies can be found in S1 Table.

### Quantification of mRNA by RT-qPCR

Liver samples were prepared as described recently [10]. Total RNA was isolated from liver tissue (approximately 8 mg) by homogenization (30 s, 6500 rpm, 4°C; Precellys-24 tissue homogenizer) in 350 μL RLT buffer (RNeasy Mini Kit) supplied with 40 mM dithiothreitol, followed by centrifugation (3 min, 16 000 × g, 4°C). The supernatant was further processed according to the manufacturer’s protocol for RNA isolation from animal tissues and cells using QIAcube. RNA quality and concentration was analyzed using Nanodrop One C. 1000 ng of RNA was transcribed into cDNA using GoScript Reverse Transcriptase. KAPA SYBR FAST Kit was used for RT-qPCR (4 ng of cDNA per reaction in triplicates, 40 cycles) analysis, and reactions were performed using a Rotor Gene Real-Time Cycler (Corbett Research, Sydney, New South Wales, Australia). Data was normalized to the expression levels of the endogenous control gene β-actin. Comparison of gene expression was performed using the 2-ΔCT-method using β-actin as housekeeping gene [67]. Primers used for RT-qPCR are listed in S2 Table.

### Extraction and quantification of miRNA by qPCR

Total RNA was extracted from liver tissues using Trizol total RNA isolation reagent and RNA concentration quantified using Nanodrop One C. TaqMan microRNA assays were used to quantify mature miR expression. SnoRNA234 was used as endogenous control of miR expression. Thus, miR-specific reverse transcription was performed for each miR using 10 ng of purified total RNA and the TaqMan MicroRNA Reverse Transcription kit according to the manufacturer’s instructions. Reactions with a volume of 15 μL were incubated for 30 min at 16°C, 30 min at 42°C, and 5 min at 85°C to inactivate the reverse transcriptase. RT-qPCR using the TaqMan Fast Advanced Master Mix and the TaqMan microRNA Assay Mix according to the manufacturer’s instructions were run in triplicates at 95°C for 10 min followed by 40 cycles at 95°C for 15 s and 60°C for 1 min. Quantitative miR expression data were acquired and analyzed using the ViiA 7 real-time PCR system.

### MicroRNA target predictions

By using the online tools TargetScan Release 7.2 (Whitehead institute, Cambridge, MA, USA, RRID:SCR_010845, [68]), RNA22 Version 2 (Thomas Jefferson University, Philadelphia, PA, USA, RRID:SCR_016507, [69]) and miRDB (MicroRNA Target Prediction Database, RRID:SCR_010848) [70], we screened the 3’-untranslated region (3’UTR) of the genes altered in the AE compared to the control group, *i*.*e*. ATF4, CHOP, ERp72, IRE1α ATF6, H6PD, GRP78 and calreticulin (S3 Table), for the presence of potential miR binding sites. The selection of the miRs was based on the study by Boubaker *et al*. [62] reporting 28 miRs with significantly altered expression levels in mice after primary *E*. *multilocularis* infection compared to non-infected mice. Referring to S3 Table, only miRs showing a potential target site in the 3’UTR of our genes of interest were analyzed by qPCR (*i*.*e*. miR-15a-5p, miR-148a-3p, miR-22-3p, miR-30a-3p, miR-30a-5p, miR-146a-5p, miR-1839-5p).

### Statistical analyses

Data are presented as mean ± SD. The significance of the differences between the examined animals were determined by Kruskal-Wallis test followed by Dunn’s Multiple Comparison post-test or one-way ANOVA followed by Bonferroni Multiple Comparison post-test, whereby the specific test is indicated in the Figure legend. No outliers were excluded. *P≤0.05; **P≤0.01; ***P≤0.001 significantly different as indicated. GaphPad Prism software (version 8.0.2, GraphPad, La Jolla, CA, USA) was used for statistical analysis.

## Results

### Mouse model of chronic *E*. *multilocularis* infection

This study employed the secondary murine AE infection model with *i*.*p*. inoculation of *E*. *multilocularis* metacestode tissue suspension, mimicking a chronic infection, and used samples from a previous investigation [10]. In this model, parasite proliferation mainly occurs in the peritoneal cavity, and the previous histopathology analysis detected parasitic structures only in some livers of infected mice but revealed immune cell infiltration in all of them [10]. The efficacy of treatment with ABZ and αPD-L1 was shown by a decreased parasite weight in the peritoneum and reduced hepatic immune cell infiltration.

### Effects of AE on the expression of proteins related to UPR and ERS pathways

**Fig 1:**
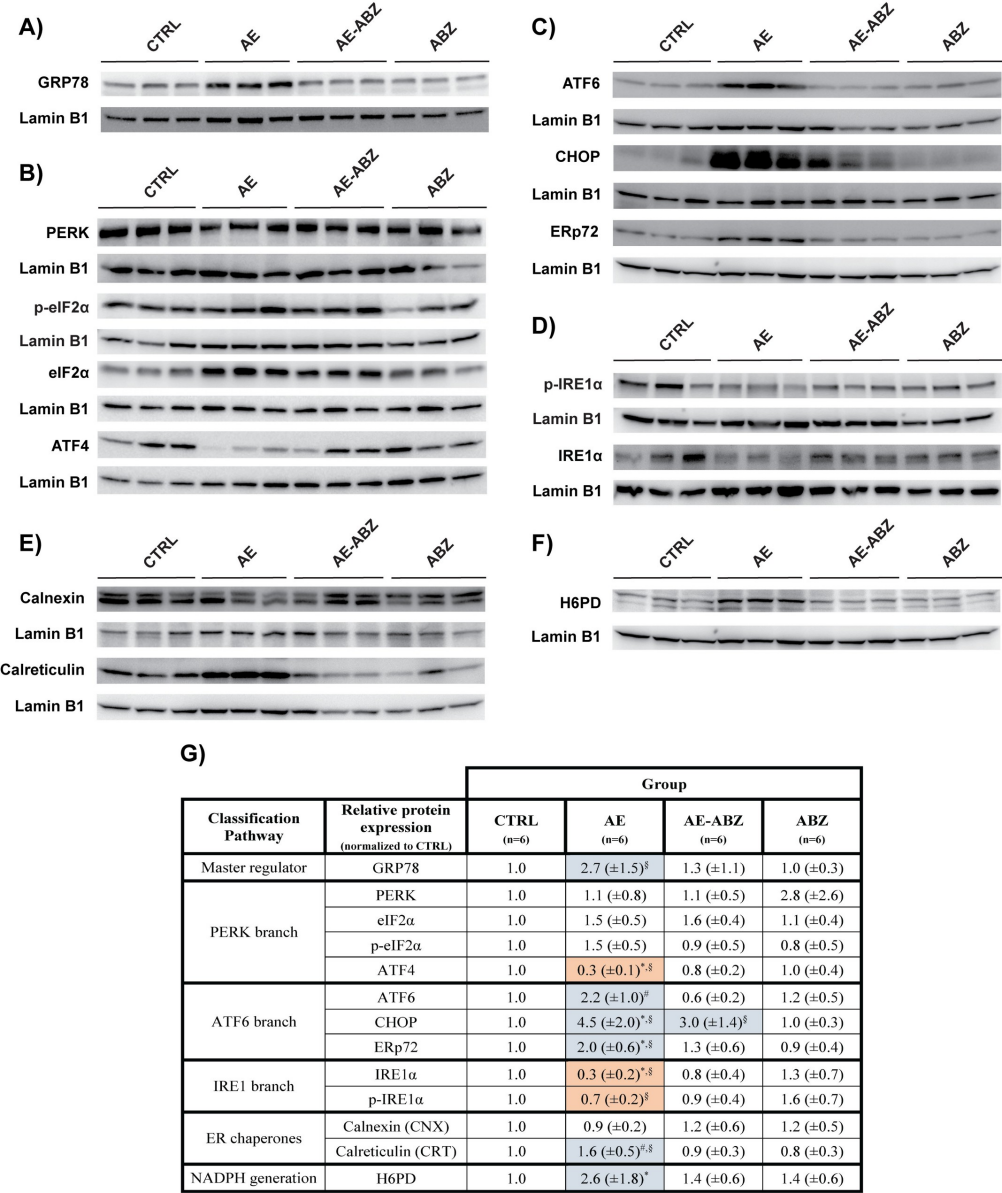
Effect of *E*. *multilocularis* infection on the expression of proteins involved in UPR and ER redox functions. Western blotting and semi-quantitative analysis by densitometry (graphs of densitometry data are shown in S2 Fig) of protein/phospho-protein levels of **A**) GRP78, **B)** PERK, eIF2α, p-eIF2α and ATF4, **C)** ATF6, CHOP, and ERp72, **D)** IRE1α and p-IRE1α, **E)** CNX and CRT, and **F)** H6PD in mock-infected control mice (CTRL), *E*. *multilocularis* infected mice (AE), infected mice treated with ABZ (AE-ABZ) or uninfected mice treated with ABZ (ABZ). One representative blot (of two) containing samples from three different mice is shown. Lamin B1 served as loading control. **G)** Bands corresponding to the respective protein/phospho-protein were analyzed by densitometry (animals per group n = 6). Numbers represent the expression of protein/phospho-protein levels normalized to those of the control (CTRL) group (mean ± SD). Significantly decreased protein/phospho-protein levels are highlighted in red and increased levels in blue. Symbols indicate significant differences (p≤0.05) between groups: *, compared to CTRL; §, compared to ABZ; #, compared to AE-ABZ. No outliers were detected/excluded. Non-parametric, Kruskal-Wallis test followed by Dunn’s Multiple Comparison post-test.

As the present knowledge on the modulation of UPR and ERS pathways by extracellular parasitic infections is limited, this study examined the expression of key proteins related to these pathways in liver tissues of mice *i*.*p*. infected with *E*. *multilocularis*. The *E*. *multilocularis* infection resulted in differential effects on the expression of proteins of the different UPR and ERS branches. GRP78, the master regulator of UPR that is common to all branches, tended to be elevated with 2.7-fold higher levels (Figs 1A, 1G and S2). Among the PERK pathway, ATF4 protein levels were significantly decreased in liver tissue of AE mice compared to mock-infected controls, whereas the expression of PERK itself and its target protein eIF2α were not affected by *E*. *multilocularis* infection (Fig 1B and 1G). Accordingly, the phosphorylation of eIF2α remained unchanged (Fig 1B–1G) while phosphorylation of PERK could not be detected. However, the most pronounced effects were observed for ERS related proteins of the ATF6 branch of the UPR (Fig 1C–1G). The levels of all three proteins analyzed were elevated in the AE group, whereby the luminal chaperone and protein disulfide isomerase ERp72 and the ERS marker CHOP were 2.0-fold and 4.5-fold increased and ATF6 was 2.2-fold higher than levels in the control group. IRE1α protein expression was decreased by about 3-fold in *E*. *multilocularis* infected compared to control mouse liver tissues and IRE1α phosphorylation tended to be lower in *E*. *multilocularis* infected mice (Fig 1D–1G). Since our available antibody was unable to properly detect XBP1 and amount of samples were limited, the expression of XBP1 and its spliced form (XBP1-s) were assessed on the mRNA level instead, which did not reveal significant differences between the treatment groups (S3 Fig).

Additional proteins with a role in ER-redox regulation and ERS include the ER resident lectin chaperones CNX and CRT. Whilst CNX protein levels were unaffected by *E*. *multilocularis* infection, CRT protein expression was significantly increased in AE mice compared to controls (Fig 1E–1G). Additionally, the expression levels of the luminal NADPH-generating enzyme H6PD were determined, revealing a 2.6-fold higher expression in AE compared to control mice (Fig 1F and 1G).

### Treatment with ABZ reverses the effects of AE on proteins involved in UPR and ERS

Treatment of AE mice with 200 mg/kg body weight ABZ (AE-ABZ group), five times per week, has been shown previously to effectively reduce parasite weight without any signs of hepatotoxicity due to drug treatment [10]. In the present study, the same treatment regimen resulted in a reversal of the *E*. *multilocularis* induced alterations of UPR and ERS related protein expression (Fig 1). Also, the effects on the ER chaperones CNX and the NADPH-generating H6PD were reversed by ABZ treatment. An exception was CHOP that was still upregulated in ABZ treated infected mice. Importantly, ABZ did not cause any significant alterations in the expression of these proteins compared to uninfected, mock-treated control mice (CTRL) (Fig 1). PERK protein levels tended to be increased in ABZ treated but uninfected animals (Fig 1); however, this did not reach significance due to high variance in the detected signals.

### Increased miR-146a-5p and miR-1839-5p expression in secondary *E*. *multilocularis* infection and reversal by ABZ treatment

Boubaker *et al*. [62], using a primary *E*. *multilocularis* infection mouse model, identified several miRs with altered expression in liver tissues from infected mice. In the present study, the miRs that were significantly altered in the study by Boubaker *et al*. [62] and that possess a potential 3’UTR binding site in at least one of the targets investigated in the present work (S3 Table) were quantified by qPCR in our secondary *E*. *mulitilocularis* infection model.

The analysis of the seven mouse miRs miR-148a-3p, miR-15a-5p, miR-22-3p, miR-146a-5p, miR-1839-5p, miR-30a-5p and miR-30a-3p revealed significantly higher levels of miR-1839-5p and miR-146a-5p in liver tissue samples of *E*. *multilocularis* infected mice (AE) compared to control animals (CTRL) (2.2-fold and 2.9 -fold, respectively, AE *vs* CTRL; S3 Fig). The other miRs remained either unchanged or showed a weak trend to be elevated (S4 Fig). Importantly, ABZ treatment reversed the elevated miR-1839-5p levels in AE mice to that seen in CTRL animals or even lower (2.0-fold and 4.1-fold, respectively, AE vs AE-ABZ). ABZ treatment in uninfected mice tended to decrease miR-1839-5p and miR-146a-5p expression levels (S3 Fig). Because miR-1839-5p is significantly increased and its predicted target IRE1α decreased in AE compared to CTRL mice, we highlighted the complementary sequence of miR-1839-5p in the 3’UTR of IRE1α (S3 Fig).

### Elevation of inflammatory cytokines due to *E*. *multilocularis* infection is reversed by ABZ treatment

**Fig 2:**
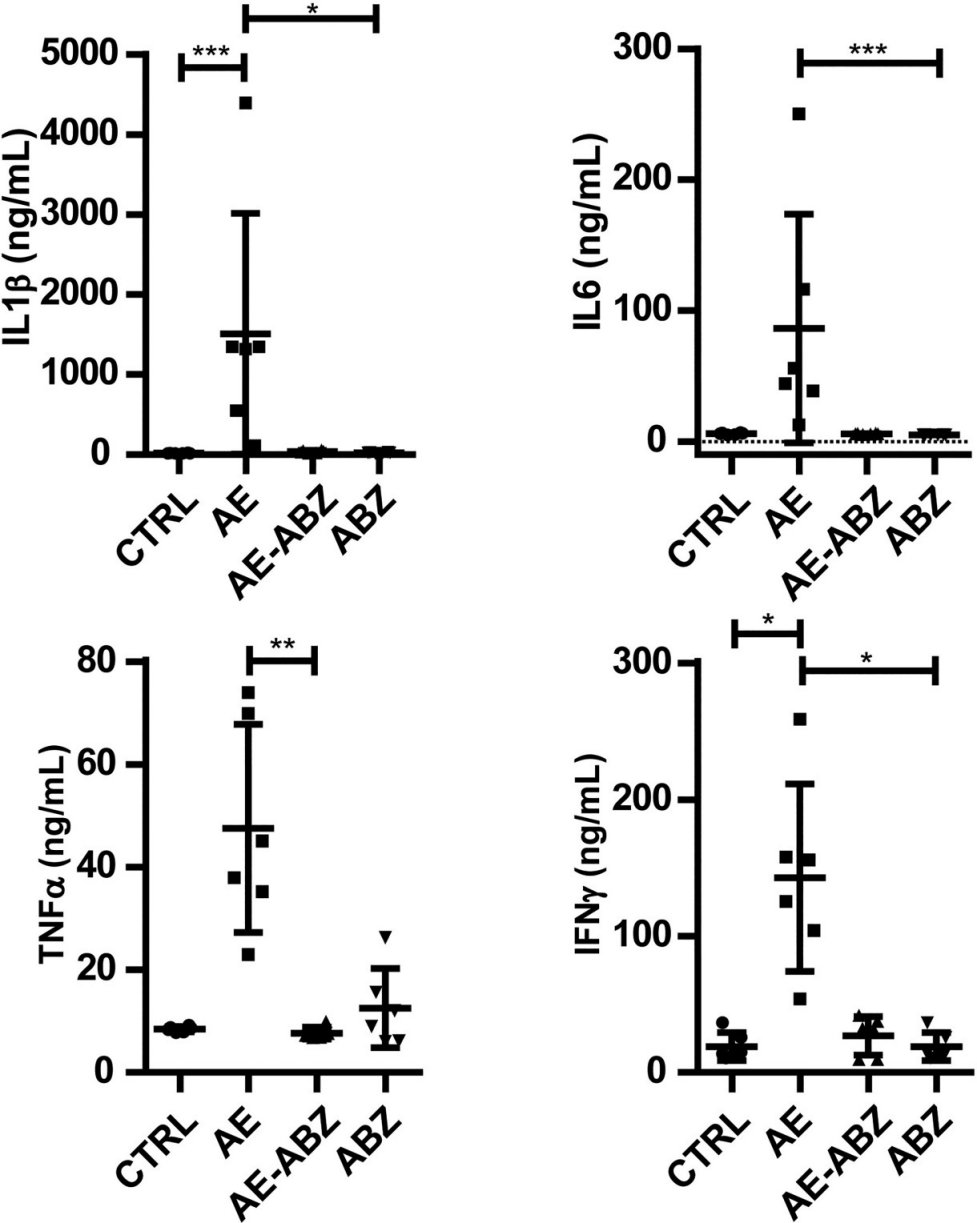
Albendazole decreases proinflammatory cytokine levels in the liver that were elevated by *E*. *multilocularis* infection. Cytokine levels of IL1β, IL6, TNFα, and IFNγ in liver tissue samples of mock-infected control mice (CTRL), *E*. *multilocularis* infected mice (AE), infected mice treated with ABZ (AE-ABZ) or uninfected mice treated with ABZ (ABZ) (animals per group n = 6). No outliers were detected/excluded. Non-parametric, Kruskal-Wallis test followed by Dunn’s Multiple Comparison post-test. *P≤0.05; **p≤0.01; ***p≤0.001.

A previous study has shown that immune modulatory treatment of AE in mice by PD-L1 blockade using antibodies successfully reduces parasite weight and inflammatory markers, such as IL-1β, IL-6, TNF-α and INF-γ [10]. In the same study, ABZ was shown to decrease the parasite weight even more than αPD-L1 treatment did, yet cytokines were not assessed. To potentially link inflammation to our current observations regarding UPR and ERS pathways, we measured the cytokines in liver samples of our present mouse model. Fig 2 shows that IL-1β, IL-6, TNF-α and INF-γ are all elevated in mice infected with *E*. *multilocularis* compared to non-infected control mice, reaching significance for IL-1β and INF-γ. Treatment with ABZ successfully reduced all the inflammatory markers back to the levels detected in non-infected control mice and itself did not alter the levels of these cytokines.

### Antibody-mediated blockade of PD-L1 reverses the effects of AE on key proteins of the UPR branches

**Fig 3:**
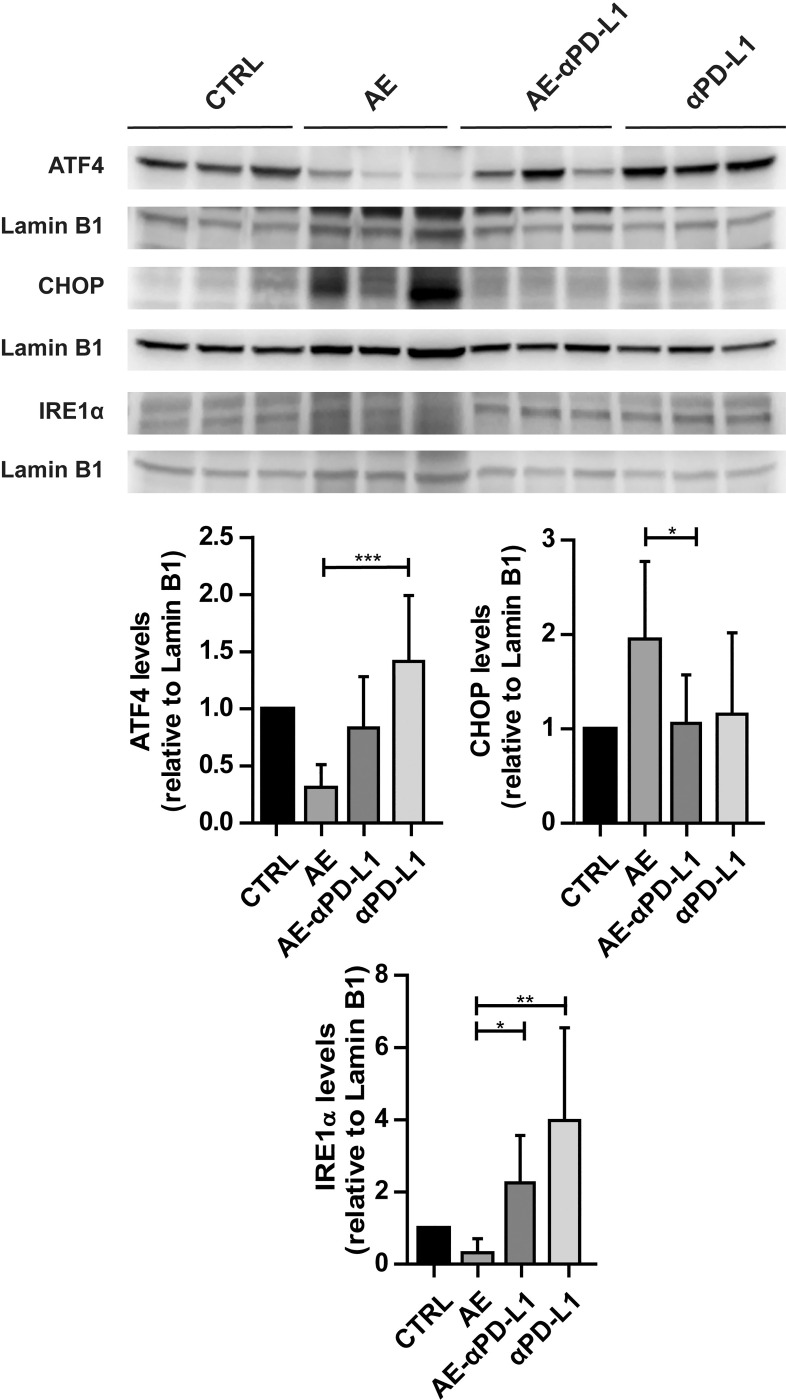
Effect of *E*. *multilocularis* infection and αPD-L1 treatment on the expression of proteins involved in UPR. Western blotting and semi-quantitative analysis by densitometry of protein levels of ATF4, CHOP, and IRE1α in mock-infected control mice (CTRL), *E*. *multilocularis* infected mice (AE), infected mice treated with αPD-L1 (AE-αPD-L1) or uninfected mice treated with αPD-L1 (αPD-L1) (animals per group n = 6). One representative blot (of two) containing samples from three different mice is shown. Lamin B1 served as loading control (animals per group n = 6). Densitometry results represent data from the two blots on samples from six mice (mean ± SD), normalized to Lamin B1 control and with CTRL set as 1. No outliers were detected/excluded. Non-parametric, Kruskal-Wallis test followed by Dunn’s Multiple Comparison post-test. *P≤0.05; **p≤0.01; ***p≤0.001.

To see whether αPD-L1 treatment ameliorates the effects of AE on UPR and ERS, we investigated the expression levels of proteins related to the three UPR branches by Western blotting. Due to the limited amount of samples, we could only investigate selected key proteins, based on the changes shown in Fig 1. The protein levels of ATF4 from the PERK branch and IRE1α from the IRE1 branch were decreased in mice infected with *E*. *multilocularis* compared to non-infected control mice (Figs 1 and 3), and αPD-L1 treatment reversed both ATF4 and IRE1α expression levels. Similarly, the increased levels of CHOP from the ATF6 branch were reversed back to levels seen in control mice.

## Discussion

**Fig 4:**
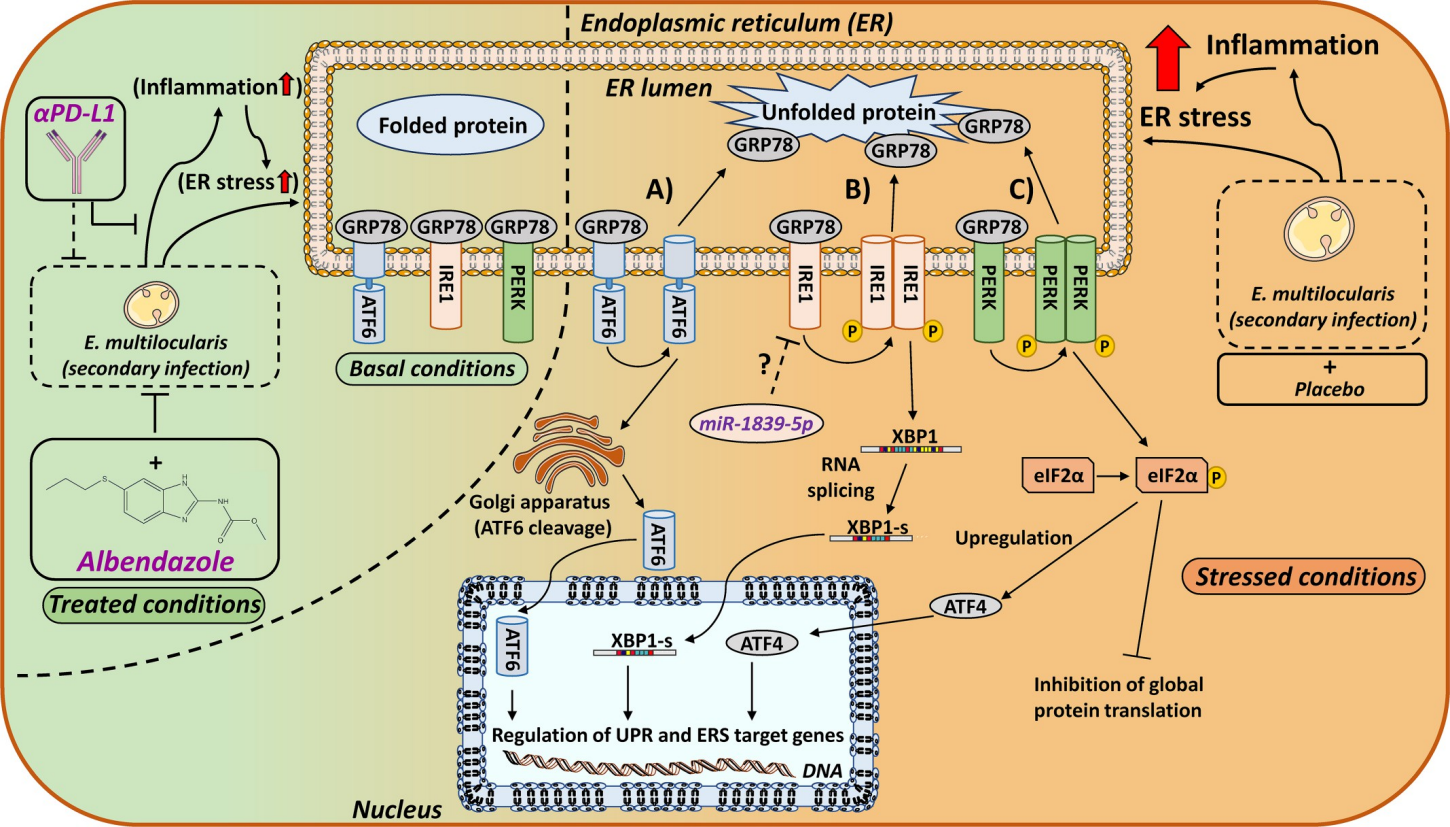
Schematic overview of ERS signaling pathways under basal and *E*. *multilocularis* infection stressed conditions. The ER chaperone GRP78 binds to unfolded luminal proteins and dissociates from the three major ERS sensors: A) ATF6, B) IRE1α and C) PERK. A) Loss of GRP78 binding leads to the translocation of ATF6 to the Golgi apparatus, where it is cleaved by proteases. The cleaved form of ATF6 translocates into the nucleus to act as a transcription factor for ER chaperons (*e*.*g*. ERp72) and ERS related genes. B) ERS promotes IRE1α dimerization and autophosphorylation, which activates the endoribonuclease activity resulting in the splicing and thereby activation of XBP1. XBP1-s promotes the expression of ERAD related genes and chaperones (*e*.*g*. GRP78). C) Activation of PERK is initiated by dimerization and self-phosphorylation. Activated PERK phosphorylates eIF2α, leading to eIF2α-mediated inhibition of global protein translation in order to decrease the luminal protein load. Besides, phosphorylated eIF2α increases the transcription of ATF4, which in turn upregulates expression of genes related to cell homeostasis restoration. If prolonged ERS occurs and pro-adaptive UPR fails, ATF4 induces genes (including CHOP) leading to apoptosis. During ERS, increased levels of miR-1839-5p potentially control the expression of the IRE1α gene, which contains a predicted target site in its 3’-UTR for this miR, thereby affecting the cellular ERS response. By suppressing the propagation of *E*. *multilocularis* infection, through yet poorly defined molecular mechanisms, ABZ and αPD-L1 treatment decrease inflammation and ERS.

Recent studies on viral, bacterial and intracellular parasitic infections emphasize the importance of the UPR and ERS pathways in pathogen-induced diseases [27–29,71,72]. Activation of the UPR, a specific form of ERS triggered by an accumulation of unfolded or misfolded proteins within the ER, can be mediated by three branches, represented by the ER transmembrane stress sensor proteins ATF6, PERK and IRE1α [73–81] (Fig 4). In non-stressed cells, these proteins remain in an inactive state, bound to the luminal chaperone GRP78. Upon activation, GRP78 is released to support luminal protein folding, followed by the activation of ATF6, PERK and IRE1α and their downstream targets such as eIF2α, ATF4, XBP1 and CHOP in order to mediate the stress responses [82–84].

Our current results revealed a pronounced induction of ATF6 in livers of mice infected with *E*. *multilocularis*. The PERK branch was less active, indicated by the downregulation of ATF4 and the unchanged protein levels of PERK, eIF2α and unchanged eIF2α phosphorylation. A phosphorylation of PERK was not detectable. The IRE1α branch also was less active since IRE1α protein and phosphorylation levels were lower or tended to be lower in *E*. *multilocularis* infected mice. The decreased ATF4 levels in livers of infected animals suggest that the observed upregulation of CHOP is mainly caused by enhanced ATF6 activity. CHOP, well-known as a mediator of apoptosis, was previously found to play an important role in the efficient expansion of the intracellular fungus *Histoplasma capsulatum* [85]. Following infection, an increase in CHOP levels led to augmented apoptosis of macrophages, thus suppressing the host’s defense and contributing to the virulence of this particular pathogen. Another study, using intestinal epithelial cell lines, showed a direct effect of heat-labile enterotoxins of *Escherichia coli* on the induction upregulation of CHOP, which led to an accelerated apoptosis of the host cells [86]. Thus, the upregulation of CHOP in murine hepatocytes during *E*. *multilocularis* infection might similarly promote parasitic growth.

In contrast to the pro-apoptotic UPR mediator CHOP, the protein levels of the PERK target ATF4 were significantly decreased in livers of *E*. *multilocularis* infected compared to mock-infected mice. This differs from a previous study on human cutaneous leishmaniasis where both CHOP and ATF4 were found to be upregulated [87]. Decreased ATF4 levels were recently described as a mechanism of acquired resistance to cope with a limited availability of amino acids in cancer cells [88]. Unrestricted tumor growth requires a high demand of nutrients and has been associated with a depletion of essential amino acids in the tumor tissue. Similar metabolic perturbations and adaptive responses may occur in patients with hepatic AE. A recent study summarizing analyses of serum samples from *E*. *multilocularis* infected and healthy adults (group size: n = 18) revealed decreased levels of branched-chain amino acids such as leucine, isoleucine and valine along with lowered levels of serine and glutamine in samples from infected patients [89]. In contrast, the aromatic amino acids tyrosine and phenylalanine were increased, together with glutamate. Thus, the observed decrease in ATF4 expression may be a response to adapt the amino acid availability in the situation of parasitic growth.

Similar to ATF4, the IRE1α protein expression and phosphorylation levels were decreased or tended to be lower in liver tissues of AE mice. The reason of the decreased IRE1α expression in *E*. *multilocularis* infected mice and the underlying mechanism remain unclear. IRE1 enzymes are transmembrane proteins exhibiting Ser/Thr protein kinase and endoribonuclease activities and acting as major ERS sensors [90,91]. There are two IRE1 isoforms in mammals: the ubiquitously expressed IRE1α and IRE1β which is predominantly expressed in the intestine and lung [92]. Further analysis of the liver resident IRE1α showed that the decreased protein expression in *E*. *multilocularis* infected mouse livers is supported by a trend of lower mRNA levels along with an increased expression of miR-1839-5p that has a target site in the 3’UTR of IRE1α as predicted by the computer-based programs Targetscan [68] and RNA22 [69]. Additionally, we found enhanced miR-146a-5p in livers of infected mice. An earlier study in primary dermal fibroblasts provided evidence for a downregulation of miR-146a-5p by IRE1-dependent cleavage in response to UPR activation [93]. Thus, the elevated miR-146a-5p may be due to decreased IRE1α activity in *E*. *multilocularis* infected mice. Furthermore, proinflammatory cytokines were found to induce miR-146a-5p [94], suggesting an upregulation of this miR in AE due to the hepatic inflammation.

An extensive analysis of miRs altered in livers of mice after primary infection with *E*. *multilocularis* by Boubaker *et al*. [62] identified several miRs with altered expression levels, including miR-1839-5p and miR-146a-5p. An increase of miR-1839-5p and miR-146a-5p in the primary as well as in the secondary infection mouse models suggests that these two miRs may represent potential biomarkers of AE; however, for this purpose they will need to be robustly detected and quantified in blood samples. In this respect, a recent study showed elevated miR-125b-5p levels in plasma of patients with AE [56], supporting the potential use of miRs as biomarkers of AE. Furthermore, Luis *et al*. reported an association of several circulating miRs, including miR-146a-5p, with ERS and organ damage in a model of trauma hemorrhagic shock [95]. Moreover, Wilczynski *et al*. reported increased miR-146a expression levels in tumor tissues of patients with ovarian cancer [96]. The advanced AE resembles a tumorigenic situation with alterations in the microenvironment and immune responses. Thus, follow-on research should address whether miR-146a-5p and miR-1839-5p can serve as serum biomarkers of AE and AE-dependent inflammation.

Besides the UPR, the ER-associated degradation (ERAD) is an important quality control machinery to cope with ER stressors. ERAD plays a crucial role in the degradation of terminally misfolded proteins by retro-translocating them from the ER to the cytoplasm for deglycosylation and ubiquitination and subsequent proteasomal degradation [97,98]. Prior to ERAD, misfolded proteins undergo repeated cycles of re-folding by the assistance of several ER-resident chaperones including lectins such as CRT and CNX, protein disulfide isomerase family members like ERp72 and ERp57 as well as members of the heat shock protein 70 family (e.g. GRP78) [99–102]. The elevated expression of CRT together with GRP78 and ERp72 indicates a higher demand for protein folding capacity in the ER in livers from infected mice. This was accompanied by an elevated demand for NADPH redox equivalents in the ER and/or an enhanced need for the products of the ER pentose phosphate pathway as indicated by the elevated H6PD expression. H6PD was found to promote cancer cell proliferation and the modulation of its expression affected GRP78, ATF6 and CHOP, emphasizing its role in ERS regulation [103].

Importantly, treatment with the parasitostatic benzimidazole ABZ and the immune-modulatory αPD-L1, which both were shown to decrease the weight of parasitic cysts in the peritoneal cavity of *i*.*p*. *E*. *multilocularis* infected mice [10], reversed the observed effects on UPR and ERS pathways and on associated ERAD and ER redox genes. Furthermore, these treatments reduced the hepatic inflammation caused by *E*. *multilocularis* infection as indicated by the reversal of the increased levels of proinflammatory cytokines in the AE-ABZ group. Our previous study, using the same infection model, showed that most mice had infiltrating parasitic structures in their liver [10]. As the concentrations of the four inflammatory cytokines showed considerable inter-individual variation, we comparatively analyzed whether this variation was associated with the presence or absence of parasitic structures in the liver, but found no correlation. Importantly, in the absence of infection, neither ABZ nor αPD-L1 affected any of the investigated ER related targets, emphasizing their favorable safety profile regarding ERS related adverse effects.

ABZ acts as an intracellular tubulin inhibitor, preventing metacestode formation [104], and it leads to a loss of integrity in the germinal layer and a reduction in metacestode mass [105]. Rodents inoculated with *E*. *multilocularis* material from ABZ treated patients, compared to inoculation with samples from untreated patients, exhibited decreased larval development [106]. At high concentrations, ABZ leads to a collapse of the alveolar architecture of the parasite, partially dissolving the laminated layer, followed by an invasion of the lesion with host inflammatory cells, such as histiocytes, lymphocytes, neutrophils and eosinophils [107]. A reduction of the width of the laminated layer upon ABZ therapy was found both in mice [108] and humans [109]. In the present study, we also observed a reduction of parasite mass.

A degradation of the laminated layer may contribute to the observed increase of small particles of *E*. *multilocularis* in and around the lesion, such as sinusoids, vessels and lymph follicles, which may influence the immune reaction [110]. Ricken *et al*. [109] showed an overall increase in the number of immune cells during the course of ABZ treatment in human AE patients. This suggests that the non-specific immune reaction is activated at the begin of ABZ treatment, with an increase in macrophages and granulocytes; and this response is shifted towards a specific immune response dominated by B and plasma cells, which does, however, not eliminate the infection. Therefore, ABZ treatment may activate the host immune system by reducing the parasite’s immunosuppressive functions. Furthermore, by reducing the metabolism of the metacestode during ABZ treatment and dissolution of the laminated layer, more parasite antigens are exposed and detected by the immune system, and this likely leads to a more specific immune response [109].

Together, this suggests that the mechanisms of ABZ and αPD-L1 are different. Inhibition of the PD-L1 pathway rather contributes to T cell activity by increasing CD4^+^/CD8^+^ effector T cells and decreasing regulatory T cells, and it has also the capacity to restore dendritic cells and Kupffer cells/macrophages and to suppresses NKT and NK cells, which leads to an improved control of *E*. *multilocularis* infection in mice.

In conclusion, the present study showed that *E*. *multilocularis* infection led to a modulation of the UPR, characterized by an activation of the ATF6-branch with an upregulation of CHOP along with decreased ATF4 and IRE1α protein levels and an increase of miR-1839-5p and miR-146a-5p. Future studies should evaluate whether these miRs can be quantified in blood samples and whether they could act as biomarkers of *E*. *multilocularis* infection and to report treatment efficacy. ABZ, the most commonly used drug to treat human AE in the clinics, as well as αPD-L1 treatment ameliorated the effects of *E*. *multilocularis* infection on ER related genes. The fact that ABZ and immune-modulatory αPD-L1 treatment both decreased the elevated levels of proinflammatory cytokines and reversed the effects of *E*. *multilocularis* infection on UPR and ERS pathways, indicates a correlation between inflammation and UPR/ERS in AE. How immune therapy and interventions in the UPR/ERS pathways could ameliorate AE warrants further investigations.

## Supporting information

S1 Table Antibodies and corresponding dilutions. (DOCX)

S2 Table Primers used for RT-qPCR. (DOCX)

S3 Table Prediction of miR target sites. (DOCX)

S1 Fig Schematic overview of the experimental setup. Animals were divided into six groups: CTRL_(n = 6)_, AE_(n = 6)_, AE-ABZ_(n = 6)_, ABZ_(n = 6)_, AE-αPD-L1_(n = 6)_ and αPD-L1_(n = 6)_. CTRL, ABZ and αPD-L1 mice received an intraperitoneal administration of 100 μL PBS. AE, AE-ABZ and AE-αPD-L1 mice were infected intraperitoneally with *E*. *multilocularis* metacestode suspension containing approximately 100 vesicular cysts resuspended in 100 μL PBS. Treatment started 6 weeks after infection. CTRL and AE mice received 100 μL corn oil orally 5 times per week and 100 μL PBS intraperitoneally twice per week for another 8 weeks. AE-ABZ and ABZ mice received ABZ (200 mg/kg body weight) in 100 μL corn oil orally 5 times per week and 100 μL PBS intraperitoneally twice per week for 8 weeks. AE-αPD-L1 and αPD-L1 mice received αPD-L1 antibody in 100 μL PBS intraperitoneally twice per week (200 μg/injection) and 100 μL corn oil orally 5 times per week. All animals were sacrificed at the end of treatment. Smart Servier Medical Art, **smart.servier.com, was used to draw the figure.**
(TIF)

S2 Fig Graphs of densitometry data of the effect of *E*. *multilocularis* infection on the expression of proteins involved in UPR and ER redox functions. Semi-quantitative analysis by densitometry of protein/phospho-protein levels of GRP78, PERK, eIF2α, p-eIF2α, and ATF4, ATF6, CHOP, and ERp72, IRE1α and p-IRE1α, calnexin, calreticulin, and H6pd in mock-infected control mice (CTRL), *E*. *multilocularis* infected mice (AE), infected mice treated with ABZ (AE-ABZ) or uninfected mice treated with ABZ (ABZ) (animals per group n = 6). Densitometry results represent data from two blots on samples from six mice (mean ± SD), normalized to Lamin B1 control and with CTRL set as 1. No outliers were detected/excluded. Non-parametric, Kruskal-Wallis test followed by Dunn’s Multiple Comparison post-test. *P≤0.05; **p≤0.01; ***p≤0.001.
(TIF)

S3 Fig IRE1α, XBP1 and XBP1-s mRNA, as well as miR-1839-5p and miR-146a-5p levels upon *E*. *multilocularis* infection and ABZ treatment. Top: IRE1α, XBP1 and XBP1-s mRNA and miR-1839-5p and miR-146a-5p levels in mock-infected control mice (CTRL _n = 6_), *E*. *multilocularis* infected mice (AE _n = 6_), infected mice treated with ABZ (AE-ABZ _n = 6_) or uninfected mice treated with ABZ (ABZ _n = 6_). mRNA levels were normalized to β-actin and miR levels to Sno234. Results represent mean ± SD. No outliers were detected/excluded. One-way ANOVA test followed by Bonferroni Multiple Comparison post-test was applied to assess significance. Bottom: Nucleotide sequence of the murine IRE1α mRNA including the 3’-UTR. The start and stop codon of the IRE1α CDS are indicated in bold and the miR-1839-5p binding site is highlighted by red and bold letters. *P≤0.05; **p≤0.01; ***p≤0.001.
(TIF)

S4 Fig *E*. *multilocularis* infection does not affect miR-15a-5p, miR-148a-3p, miR-22-3p, miR-30a-3p and miR-30a-5p expression levels. miR-15a-5p, miR-148a-3p, miR-22-3p, miR-30a-5p and miR-30a-3p levels, in mock-infected, mock-treated mice (CTRL _n = 6_) and *E*. *multilocularis* infected mock-treated mice (AE _n = 6_). Results represent mean ± SD. No outliers were excluded. Two-tailed unpaired t-test was applied to test significance.
(TIF)

S1 File Raw data of Western blotting used to produce graphs and figures. (PDF)
