# Supplementary material for: Albendazole reduces hepatic inflammation and endoplasmic reticulum-stress in a mouse model of chronic Echinococcus multilocularis infection
Source: PLoS Negl Trop Dis. 2022 Jan 14;16(1):e0009192. doi: 10.1371/journal.pntd.0009192 (PMC8794265; doi:10.1371/journal.pntd.0009192)
Supplement: S1 Table — (DOCX) [file pntd.0009192.s001.docx]

**S1 Table. Antibodies and corresponding dilutions**

| **Primary antibodies (ab)** | | | | |
| --- | --- | --- | --- | --- |
| **Target protein** | **Species** | **Type** | **Dilutions** | |
|  |  |  | **Primary ab** | **Secondary ab** |
| H6PD | Rabbit | Polyclonal | 1:1000 | 1:2000 |
| CRT | Rabbit | Polyclonal | 1:1000 | 1:2000 |
| CNX | Rabbit | Polyclonal | 1:1000 | 1:2000 |
| GRP78 | Mouse | Monoclonal | 1:1000 | 1:4000 |
| CHOP | Mouse | Monoclonal | 1:1000 | 1:4000 |
| ATF4 | Rabbit | Monoclonal | 1:1000 | 1:2000 |
| ATF6 | Rabbit | Monoclonal | 1:1000 | 1:2000 |
| ERp72 | Mouse | Monoclonal | 1:1000 | 1:4000 |
| IRE1α | Mouse | Monoclonal | 1:1000 | 1:4000 |
| p-IRE1α | Rabbit | Polyclonal | 1:1000 | 1:2000 |
| PERK | Mouse | Monoclonal | 1:1000 | 1:4000 |
| p-PERK | Rabbit | Monoclonal | 1:1000 | 1:2000 |
| eIF2α | Rabbit | Polyclonal | 1:1000 | 1:4000 |
| p-eIF2α | Rabbit | Monoclonal | 1:1000 | 1:2000 |
| Lamin B1 | Rabbit | Monoclonal | 1:1000 | 1:2000 |
