# Supplementary material for: Albendazole reduces hepatic inflammation and endoplasmic reticulum-stress in a mouse model of chronic Echinococcus multilocularis infection
Source: PLoS Negl Trop Dis. 2022 Jan 14;16(1):e0009192. doi: 10.1371/journal.pntd.0009192 (PMC8794265; doi:10.1371/journal.pntd.0009192)
Supplement: S2 Table — (DOCX) [file pntd.0009192.s002.docx]

**S2 Table. Primers used for RT-qPCR**

| **Primers** | | |
| --- | --- | --- |
| **Target gene** | **Forward primer** | **Reverse primer** |
| *IRE1α* | 5’-TGTGGTCAAGATGGACTGGC-3’ | 5’-TCGGAGGAGGTCTCTCACAG-3’ |
| *Xbp1-s* | 5′-GAGTCCGCAGCAGGTG-3′ | 5′-GTGTCAGAGTCCATGGGA-3′ |
| *Xbp1* | 5′-AAGAACACGCTTGGGAATGG-3′ | 5′-ACTCCCCTTGGCCTCCAC-3′ |
| *β-Actin* | 5’-ACCCTGTGCTGCTCACCGA-3’ | 5’-CTGGATGGCTACGTACATGGCT-3’ |
