# Supplementary material for: Albendazole reduces hepatic inflammation and endoplasmic reticulum-stress in a mouse model of chronic Echinococcus multilocularis infection
Source: PLoS Negl Trop Dis. 2022 Jan 14;16(1):e0009192. doi: 10.1371/journal.pntd.0009192 (PMC8794265; doi:10.1371/journal.pntd.0009192)
Supplement: S3 Table — (DOCX) [file pntd.0009192.s003.docx]

| **Classification**  **Pathway** | **Protein** | **Gene** | **MicroRNA** | **Sites in 3’UTR** | **Software** |
| --- | --- | --- | --- | --- | --- |
| Master regulator | GRP78 | *Hspa5* | mmu-miR-30a-5p | 1 | Targetscan |
|  |  |  | mmu-miR-148a-3p | 1 | miRDB |
| PERK branch | ATF4 | *Atf4* | None | None | None |
| ATF6 branch | ATF6 | *Atf6* | mmu-miR-15a-5p | 1 | Targetscan, miRDB |
|  |  |  | mmu-miR-146a-5p | 3 | Targetscan |
|  |  |  | mmu-miR-148a-3p | 1 | Targetscan |
|  |  |  | mmu-miR-1839-5p | 1 | Targetscan |
|  | CHOP | *Ddit3* | mmu-miR-148a-3p | 2 | Targetscan, miRDB |
|  | ERp72 | *Pdia4* | mmu-miR-30a-3p | 1 | miRDB |
| IRE1 branch | IRE1α | *Ern1* | mmu-miR-15a-5p | 2 | Targetscan |
|  |  |  | mmu-miR-22-3p | 1 | RNA22, Targetscan |
|  |  |  | mmu-miR-30a-3p | 1 | miRDB |
|  |  |  | mmu-miR-148a-3p | 1 | RNA22, Targetscan |
|  |  |  | mmu-miR-1839-5p | 1 | RNA22, Targetscan |
| ER chaperone | Calreticulin | *Calr* | mmu-miR-22-3p | 1 | RNA22 |
|  |  |  | mmu-miR-148a-3p | 1 | Targetscan |
|  |  |  | mmu-miR-1839-5p | 1 | RNA22 |
| NADPH generation | H6PD | *H6pd* | mmu-miR-22-3p | 2 | Targetscan |
|  |  |  | mmu-miR-30a-3p | 1 | miRDB |

**S3 Table. Prediction of miRNA target sites.**
