# Supplementary figures and images for: Albendazole reduces hepatic inflammation and endoplasmic reticulum-stress in a mouse model of chronic Echinococcus multilocularis infection
Source: PLoS Negl Trop Dis. 2022 Jan 14;16(1):e0009192. doi: 10.1371/journal.pntd.0009192 (PMC8794265; doi:10.1371/journal.pntd.0009192)

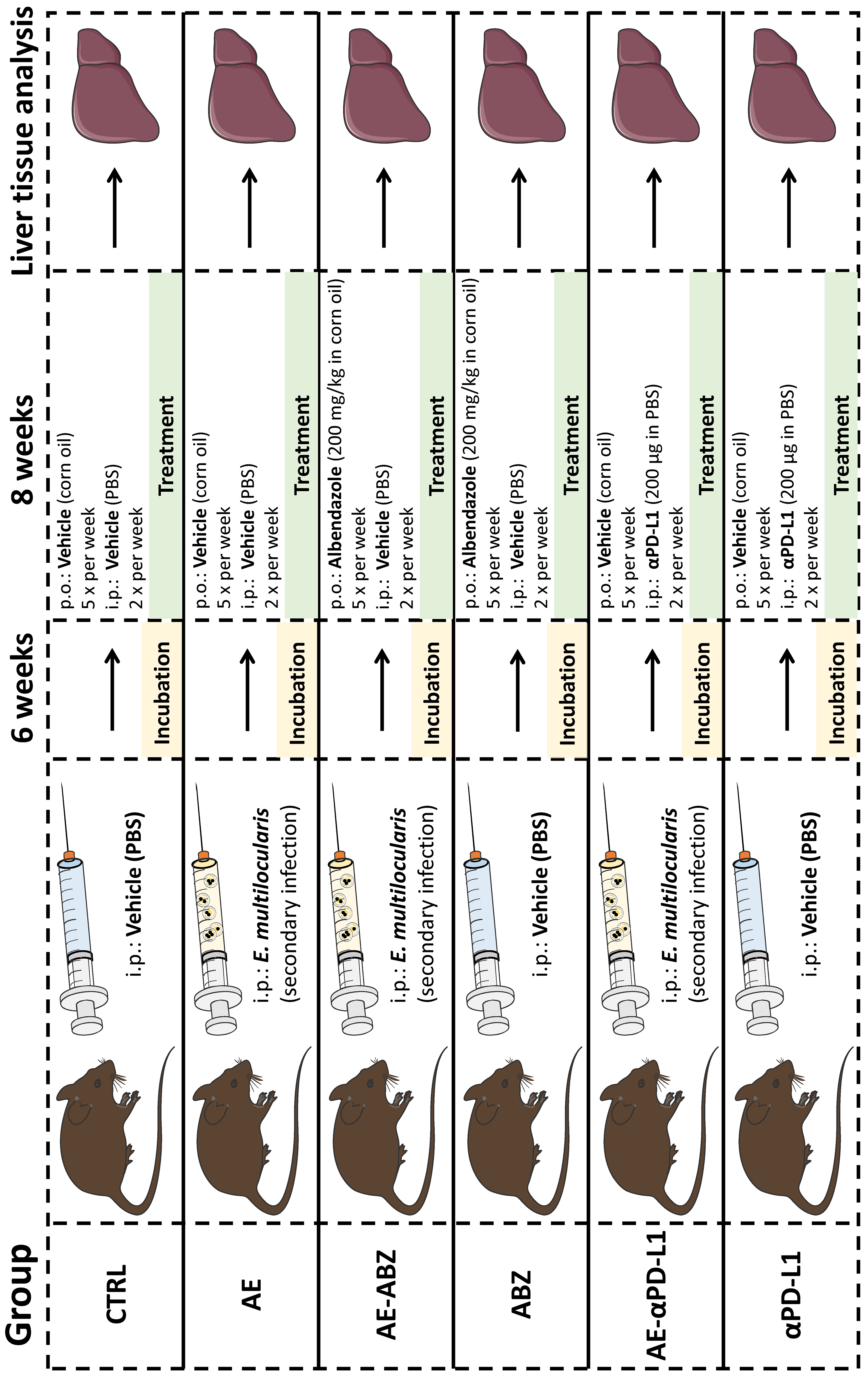

Supplement: S1 Fig — Animals were divided into six groups: CTRL(n = 6), AE(n = 6), AE-ABZ(n = 6), ABZ(n = 6), AE-αPD-L1(n = 6) and αPD-L1(n = 6). CTRL, ABZ and αPD-L1 mice received an intraperitoneal administration of 100 μL PBS. AE, AE-ABZ and AE-αPD-L1 mice were infected intraperitoneally with E. multilocularis metacestode suspension containing approximately 100 vesicular cysts resuspended in 100 μL PBS. Treatment started 6 weeks after infection. CTRL and AE mice received 100 μL corn oil orally 5 times per week and 100 μL PBS intraperitoneally twice per week for another 8 weeks. AE-ABZ and ABZ mice received ABZ (200 mg/kg body weight) in 100 μL corn oil orally 5 times per week and 100 μL PBS intraperitoneally twice per week for 8 weeks. AE-αPD-L1 and αPD-L1 mice received αPD-L1 antibody in 100 μL PBS intraperitoneally twice per week (200 μg/injection) and 100 μL corn oil orally 5 times per week. All animals were sacrificed at the end of treatment. Smart Servier Medical Art, smart.servier.com, was used to draw the figure. (TIF) [file pntd.0009192.s004.tif]

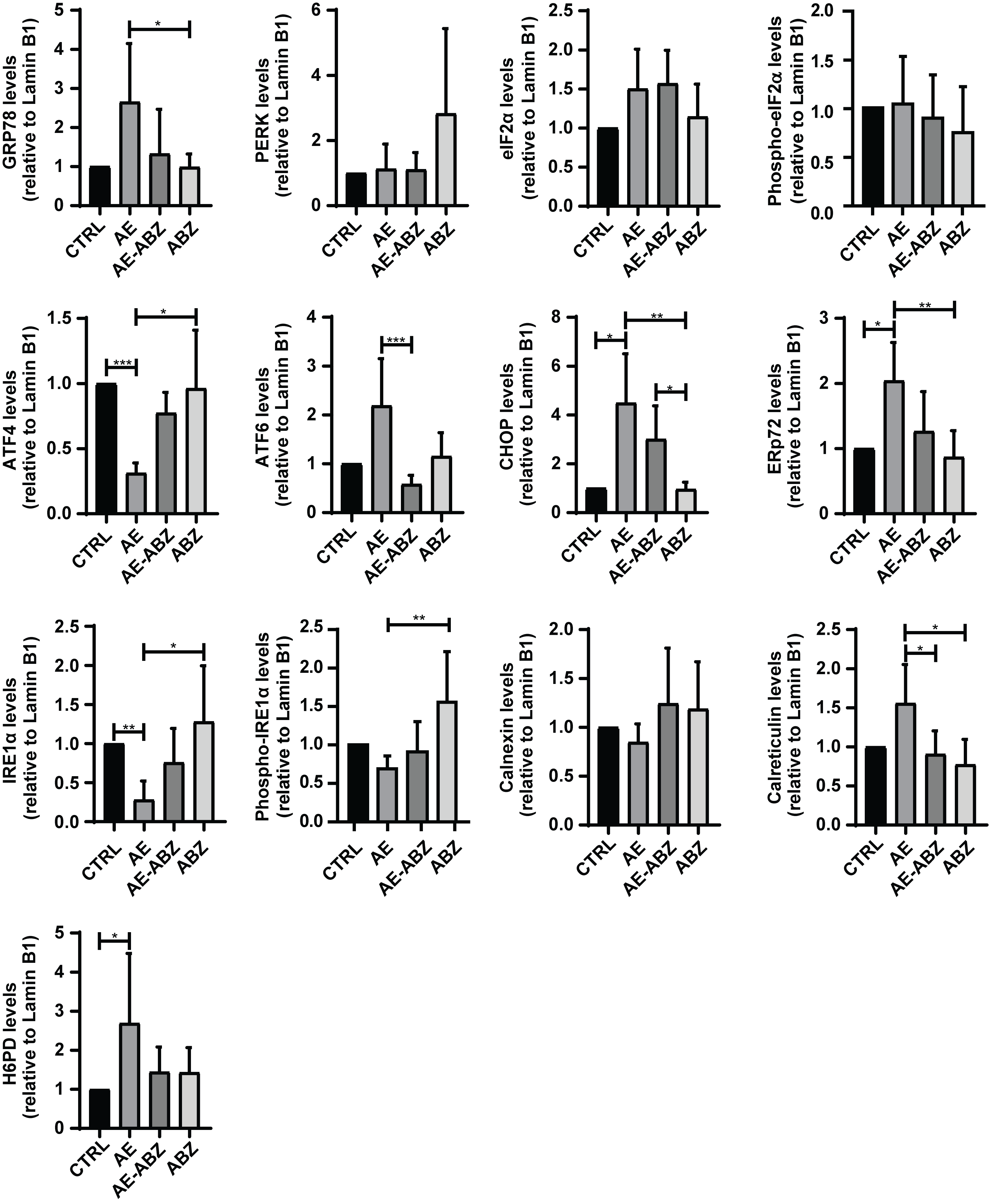

Supplement: S2 Fig — Semi-quantitative analysis by densitometry of protein/phospho-protein levels of GRP78, PERK, eIF2α, p-eIF2α, and ATF4, ATF6, CHOP, and ERp72, IRE1α and p-IRE1α, calnexin, calreticulin, and H6pd in mock-infected control mice (CTRL), E. multilocularis infected mice (AE), infected mice treated with ABZ (AE-ABZ) or uninfected mice treated with ABZ (ABZ) (animals per group n = 6). Densitometry results represent data from two blots on samples from six mice (mean ± SD), normalized to Lamin B1 control and with CTRL set as 1. No outliers were detected/excluded. Non-parametric, Kruskal-Wallis test followed by Dunn’s Multiple Comparison post-test. *P≤0.05; **p≤0.01; ***p≤0.001. (TIF) [file pntd.0009192.s005.tif]

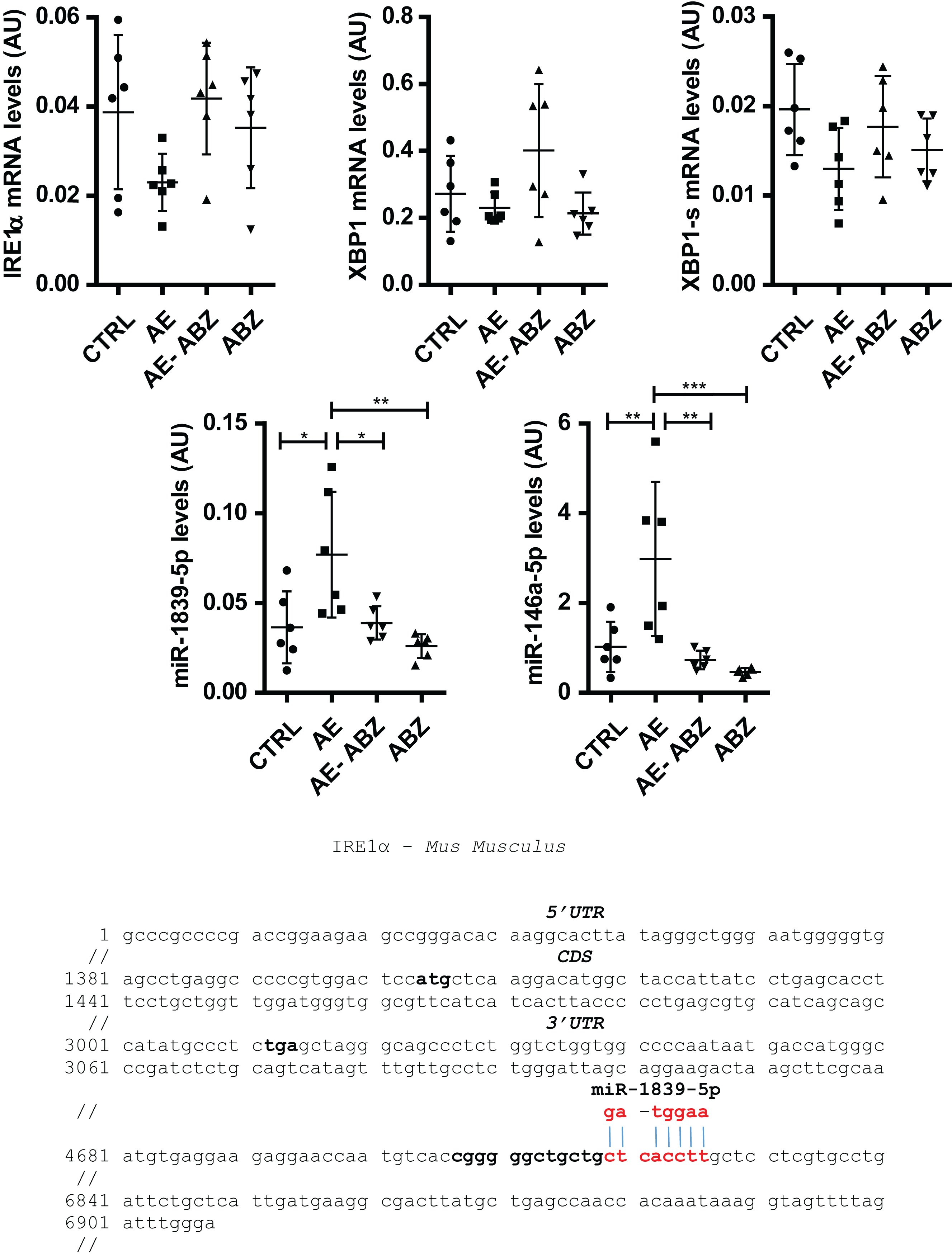

Supplement: S3 Fig — Top: IRE1α, XBP1 and XBP1-s mRNA and miR-1839-5p and miR-146a-5p levels in mock-infected control mice (CTRL n = 6), E. multilocularis infected mice (AE n = 6), infected mice treated with ABZ (AE-ABZ n = 6) or uninfected mice treated with ABZ (ABZ n = 6). mRNA levels were normalized to β-actin and miR levels to Sno234. Results represent mean ± SD. No outliers were detected/excluded. One-way ANOVA test followed by Bonferroni Multiple Comparison post-test was applied to assess significance. Bottom: Nucleotide sequence of the murine IRE1α mRNA including the 3’-UTR. The start and stop codon of the IRE1α CDS are indicated in bold and the miR-1839-5p binding site is highlighted by red and bold letters. *P≤0.05; **p≤0.01; ***p≤0.001. (TIF) [file pntd.0009192.s006.tif]

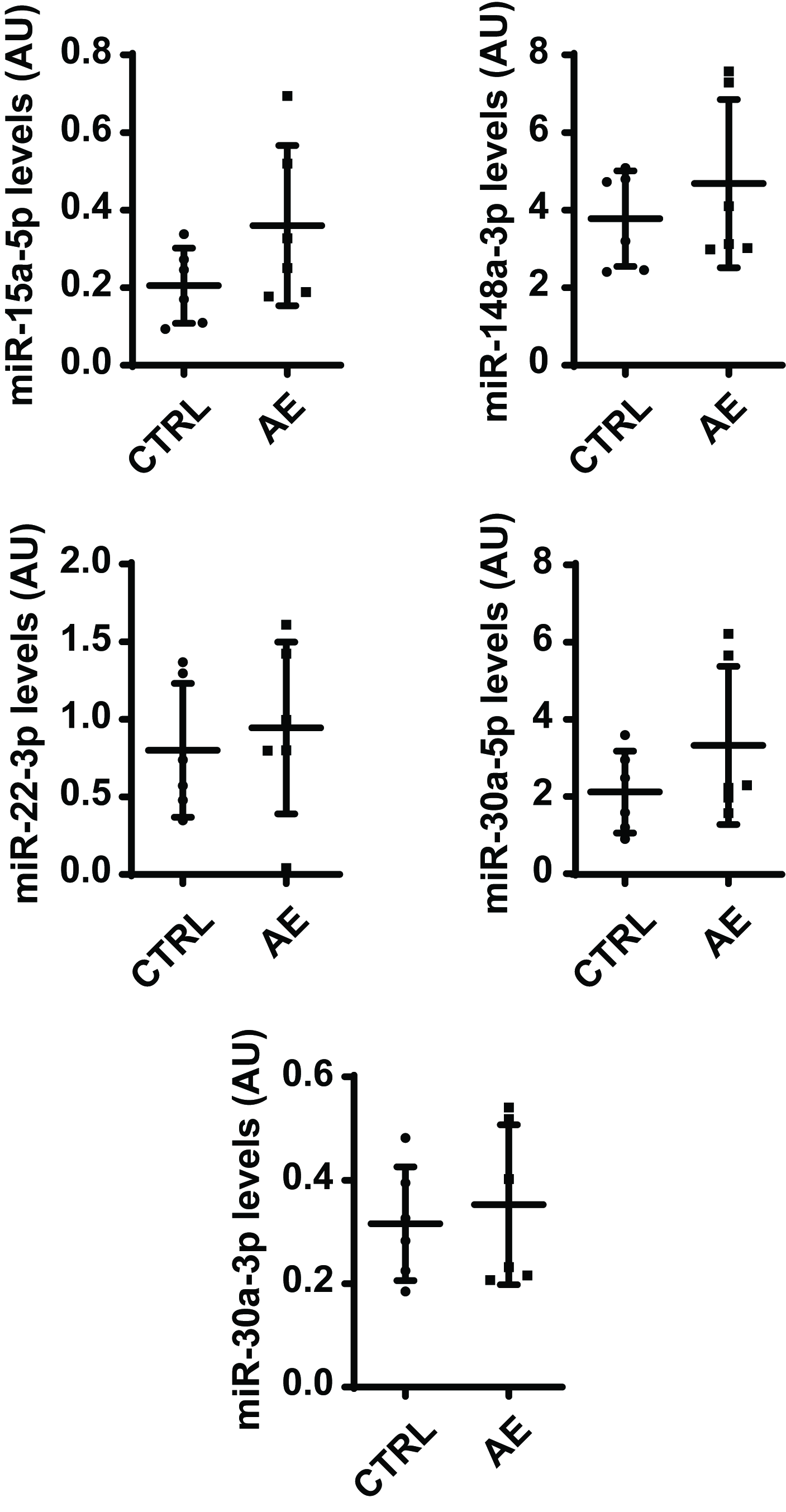

Supplement: S4 Fig — miR-15a-5p, miR-148a-3p, miR-22-3p, miR-30a-5p and miR-30a-3p levels, in mock-infected, mock-treated mice (CTRL n = 6) and E. multilocularis infected mock-treated mice (AE n = 6). Results represent mean ± SD. No outliers were excluded. Two-tailed unpaired t-test was applied to test significance. (TIF) [file pntd.0009192.s007.tif]
