# Supplementary material for: Albendazole reduces hepatic inflammation and endoplasmic reticulum-stress in a mouse model of chronic Echinococcus multilocularis infection
Source: PLoS Negl Trop Dis. 2022 Jan 14;16(1):e0009192. doi: 10.1371/journal.pntd.0009192 (PMC8794265; doi:10.1371/journal.pntd.0009192)

S1 File - Raw blots

Numbering:

I: CTRL, AE, AE-ABZ, ABZ; animals 1-3

II: CTRL, AE, AE-ABZ, ABZ; animals 4-6

III: CTRL, AE, AE-αPD-L1, αPD-L1; animals 1-3

IV: CTRL, AE, AE-αPD-L1, αPD-L1; animals 4-6

Note: As a result of limited sample amount the authors partially reused the presented western blots for the detection of different target proteins.

GRP78 and the associated loading control Lamin B1 – I:

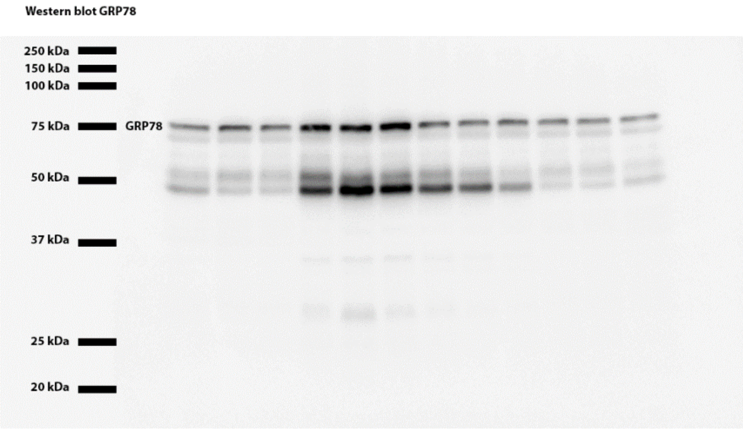

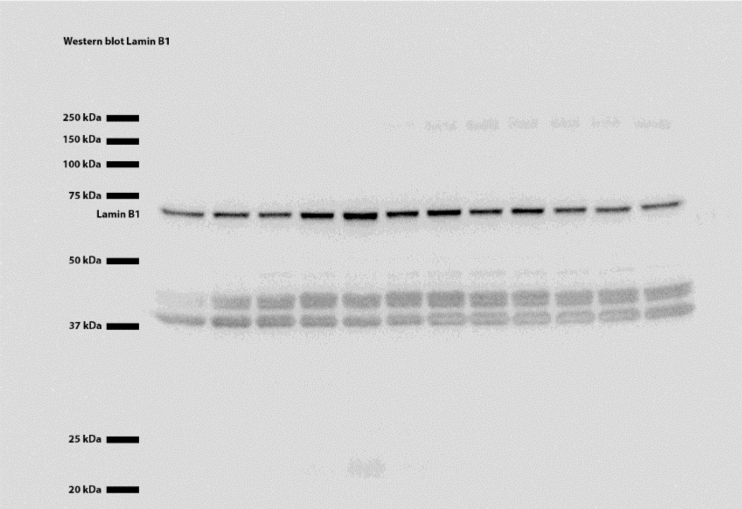

GRP78 and the associated loading control Lamin B1 – II:

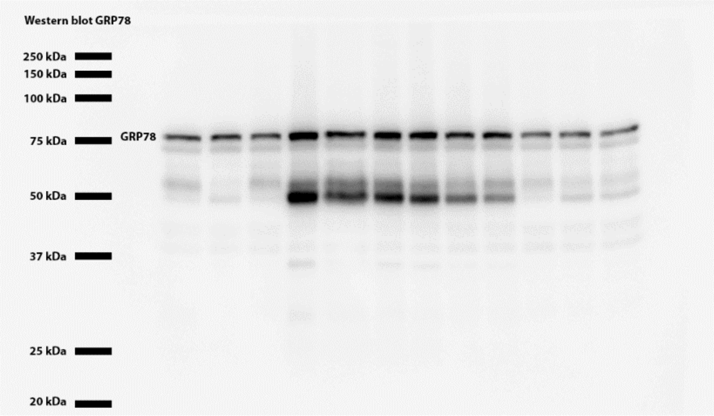

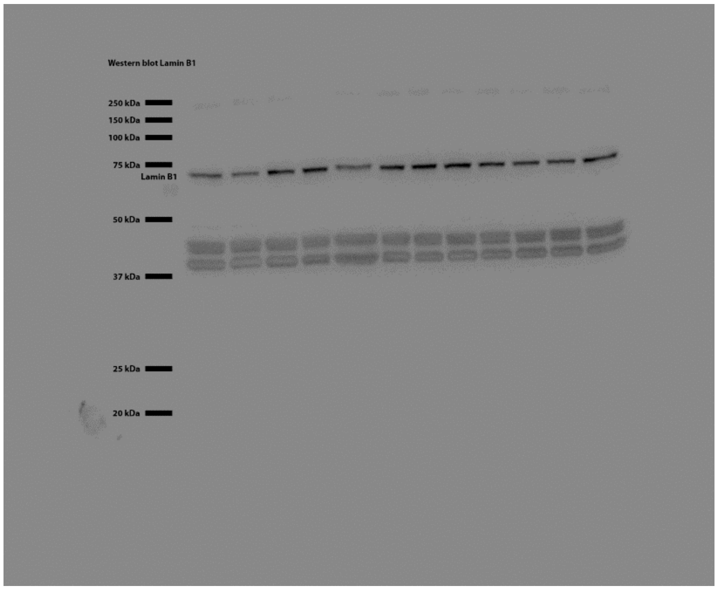

PERK and the associated loading control Lamin B1 – I:

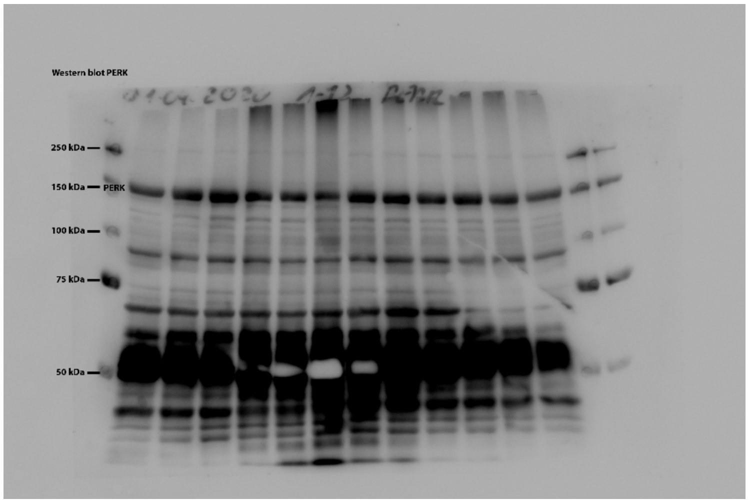

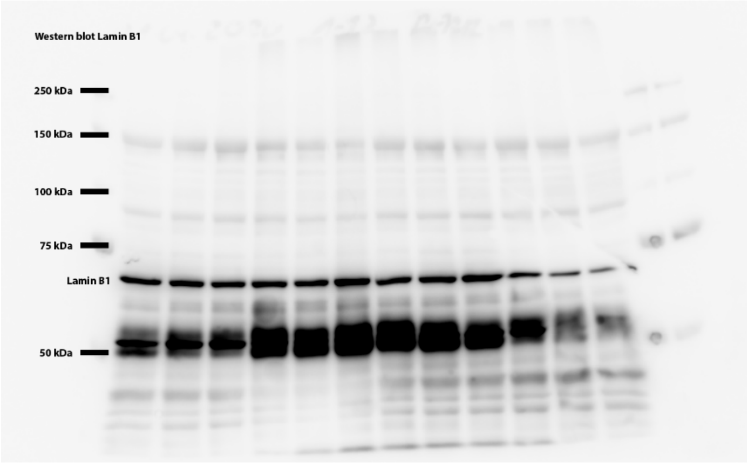

PERK and the associated loading control Lamin B1 – II:

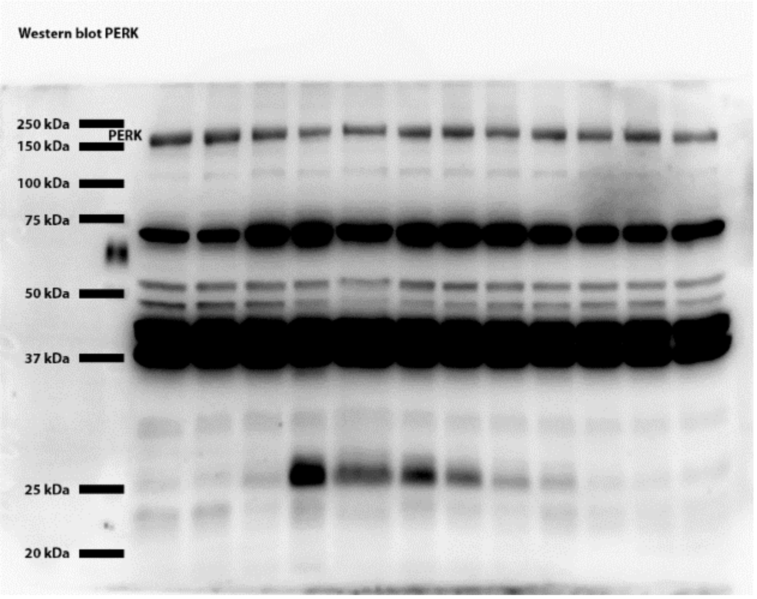

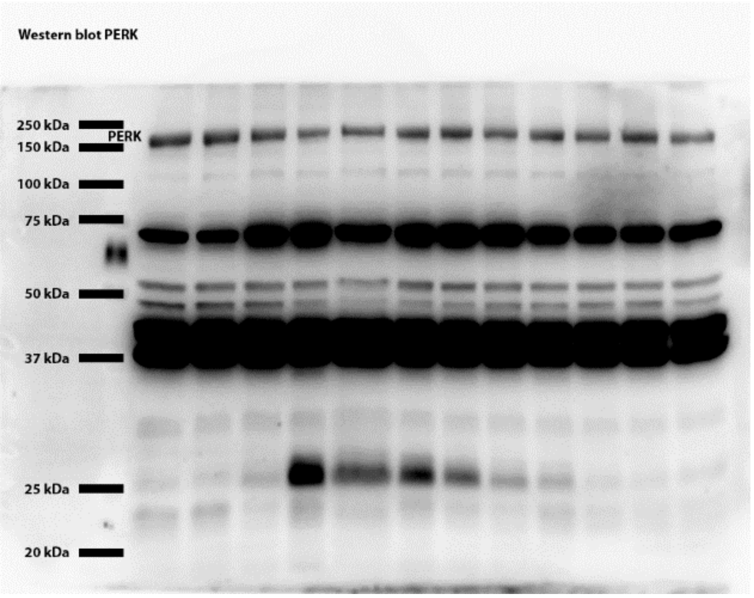

eIF2α and the associated loading control Lamin B1 – I:

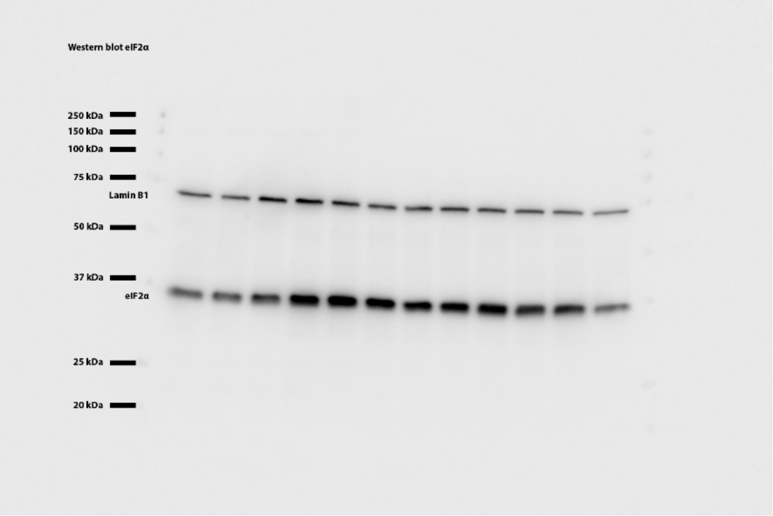

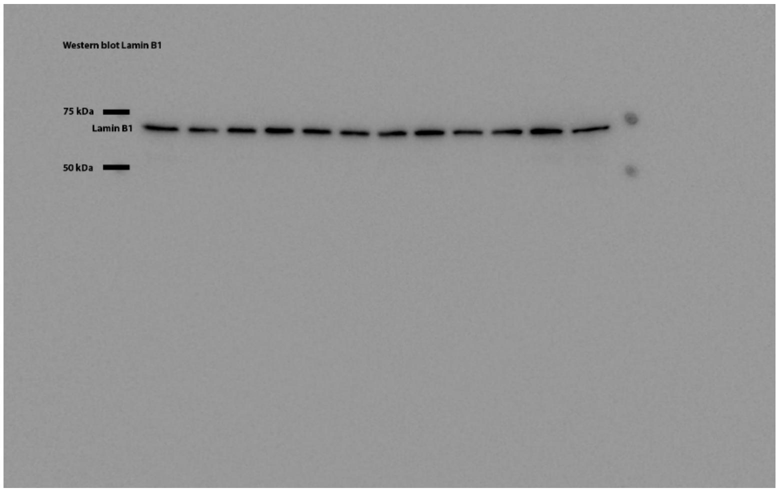

eIF2α and the associated loading control Lamin B1 – II:

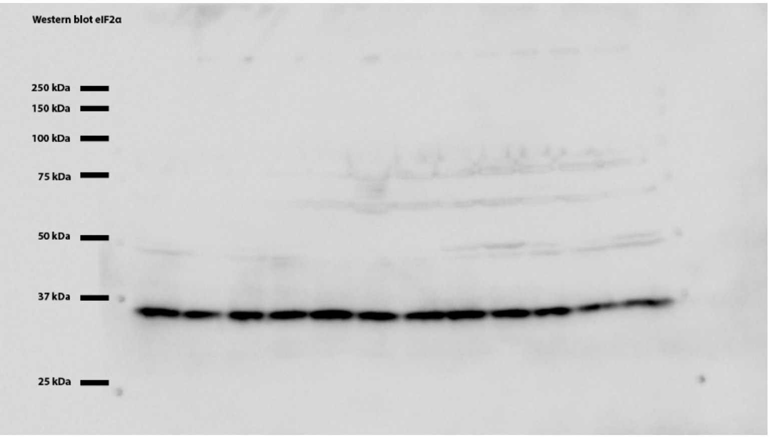

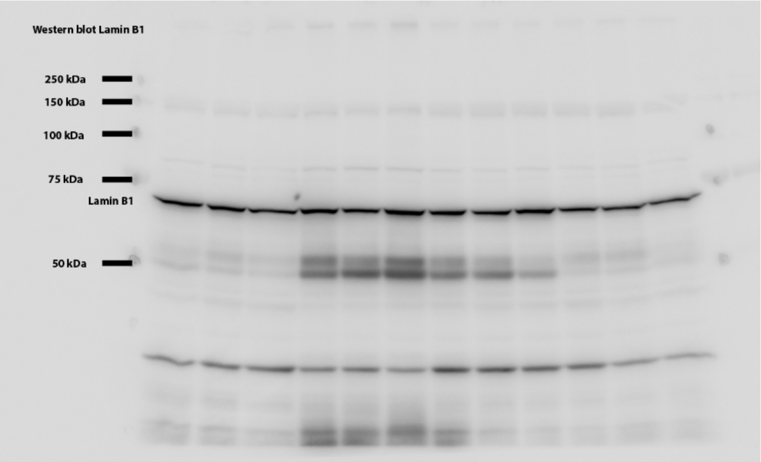

p-eIF2α and the associated loading control Lamin B1 – I:

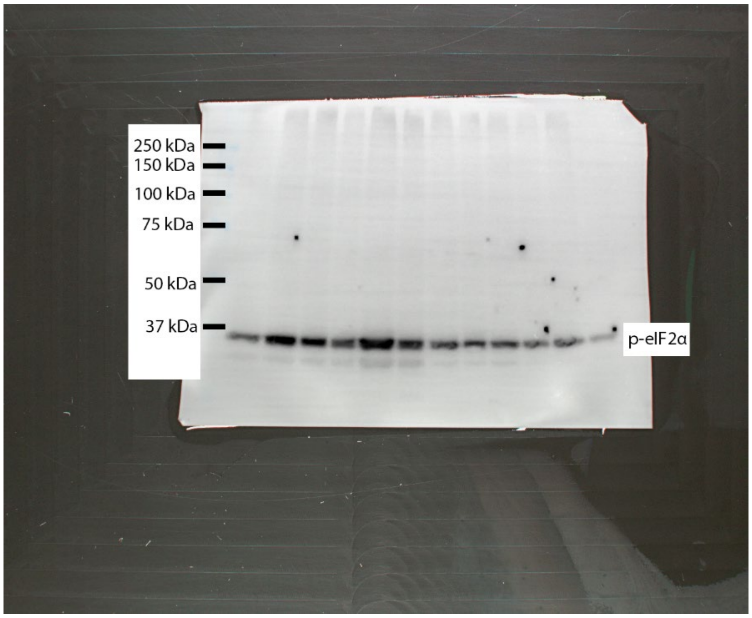

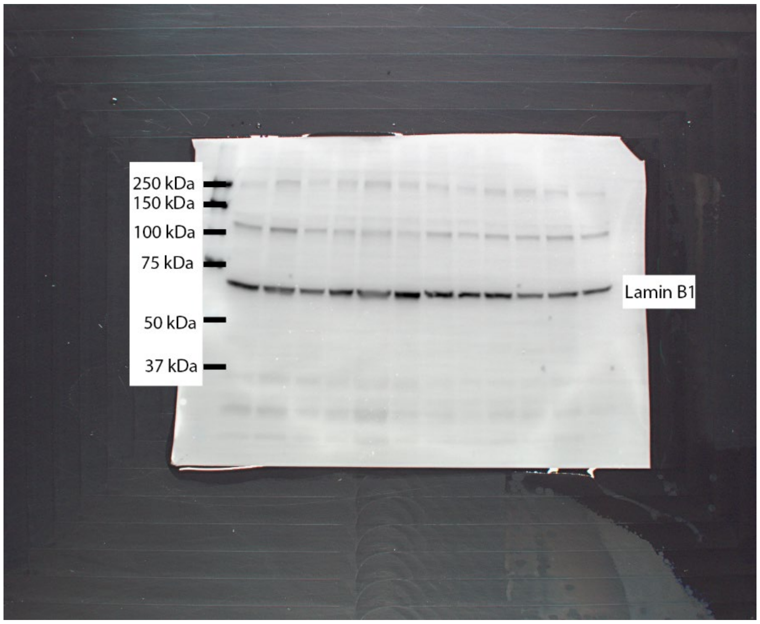

p-eIF2α and the associated loading control Lamin B1 – II:

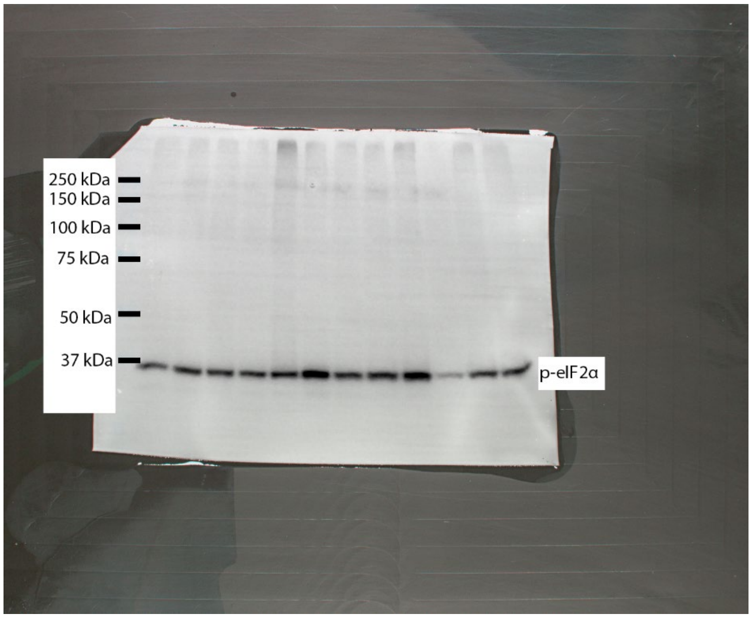

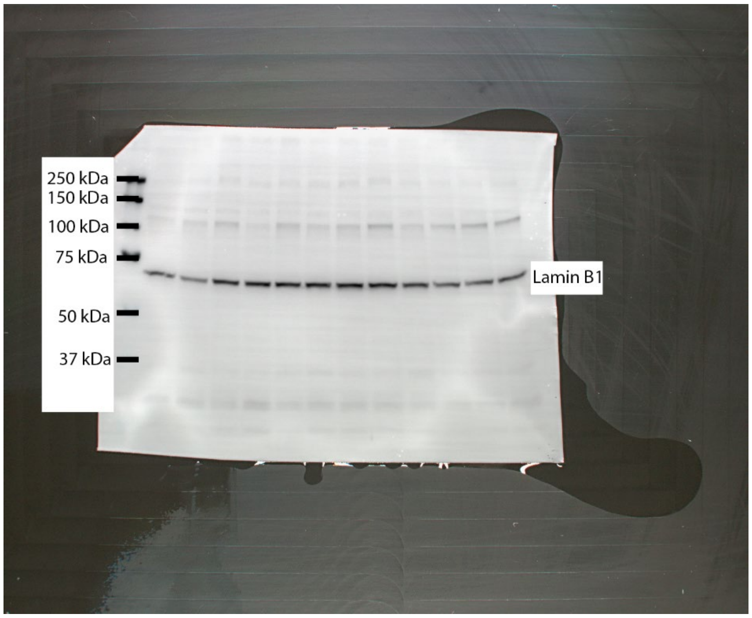

ATF4 and the associated loading control Lamin B1 – I:

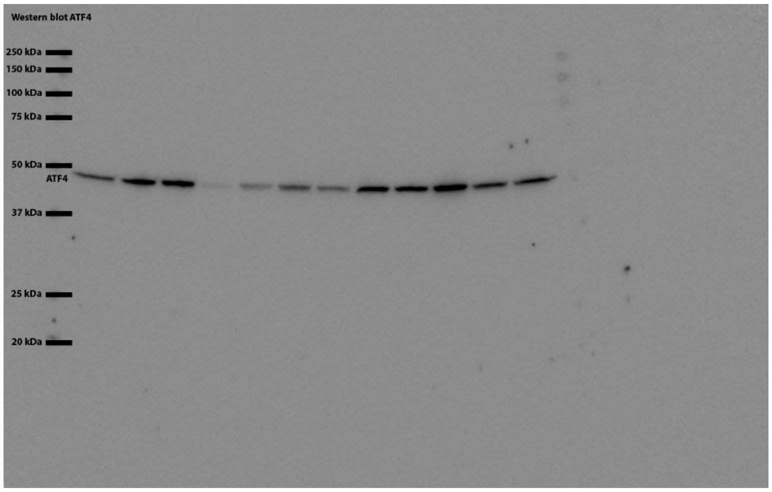

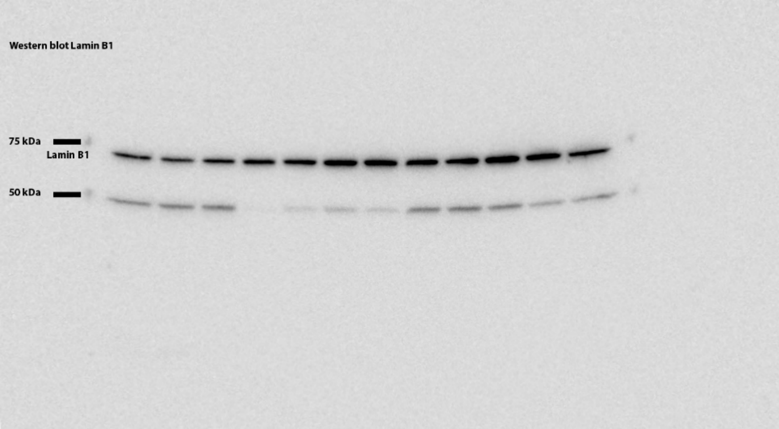

ATF4 and the associated loading control Lamin B1 – II:

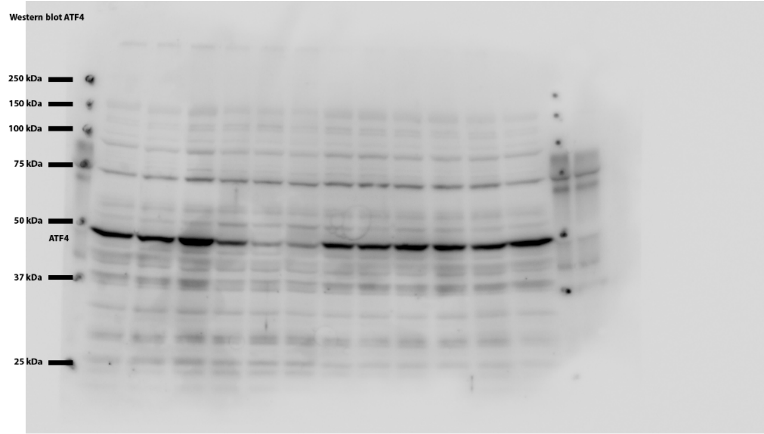

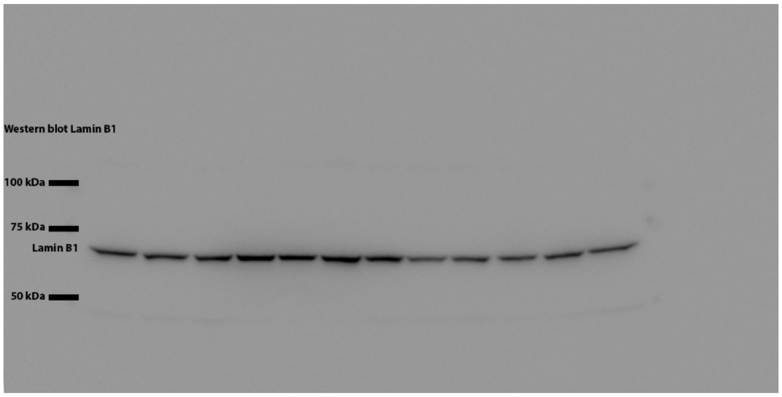

ATF4 and the associated loading control Lamin B1 – III:

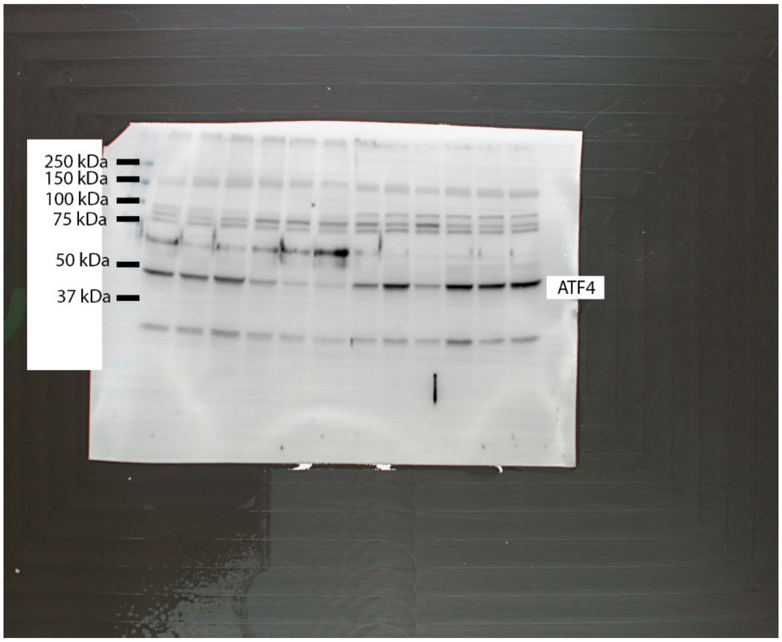

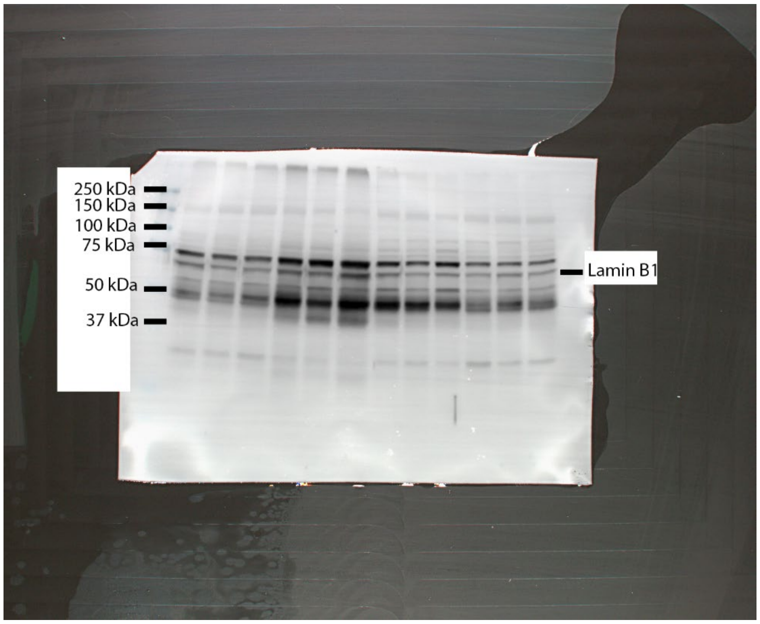

ATF4 and the associated loading control Lamin B1 – IV:

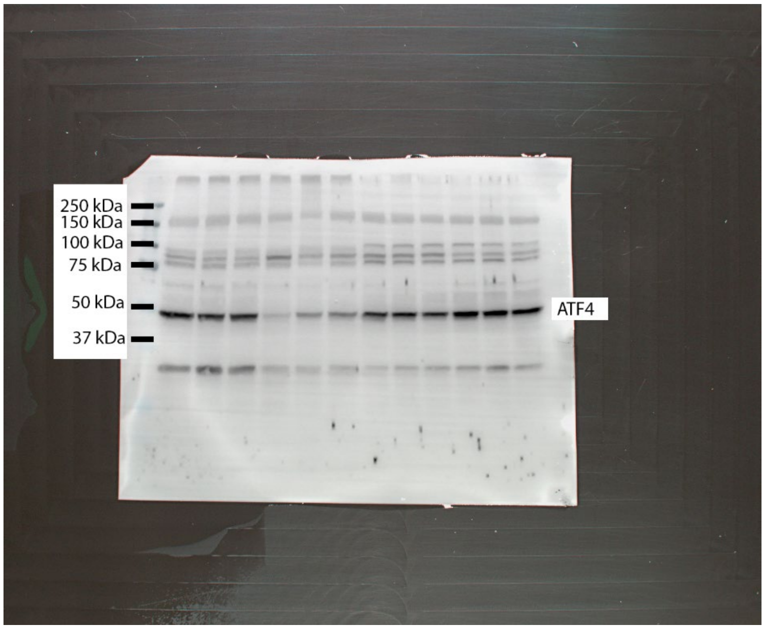

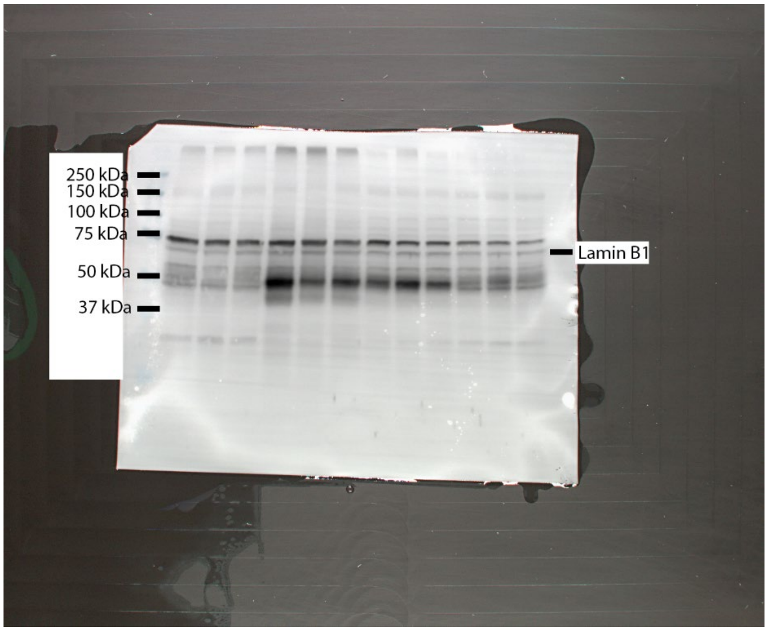

ATF6 and the associated loading control Lamin B1 – I:

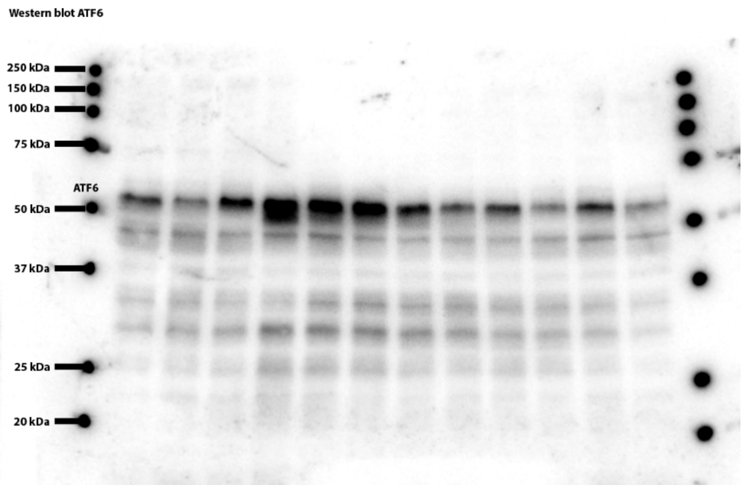

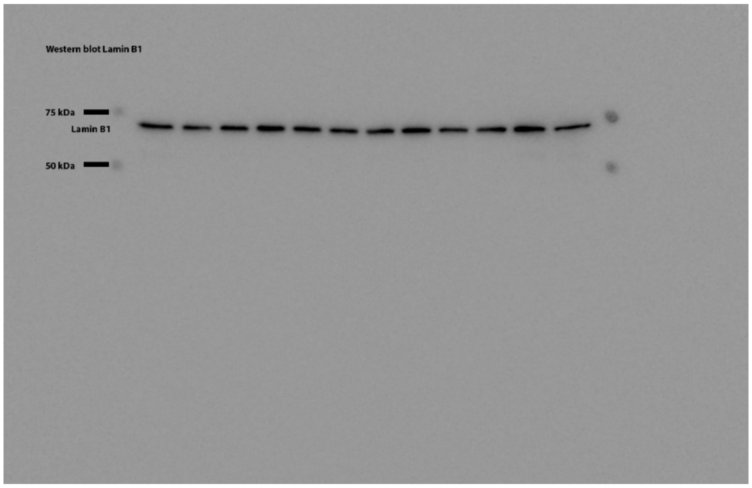

ATF6 and the associated loading control Lamin B1 – II:

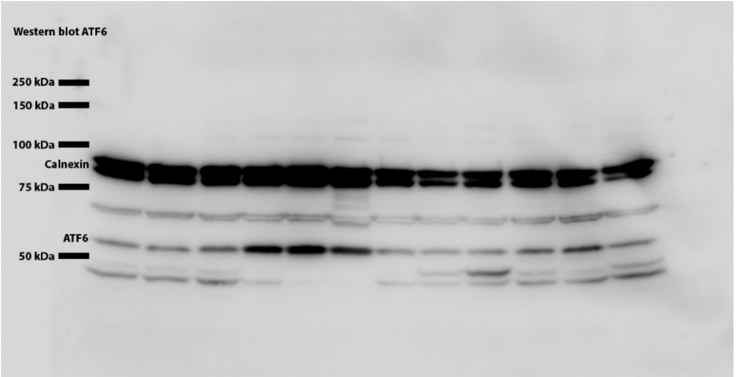

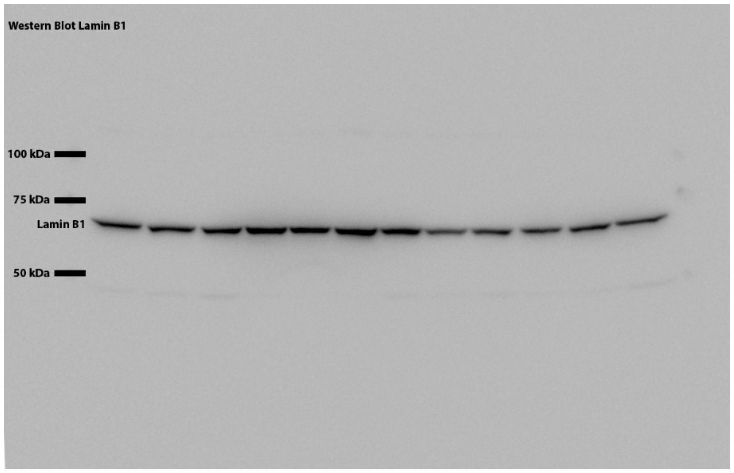

CHOP and the associated loading control Lamin B1 – I:

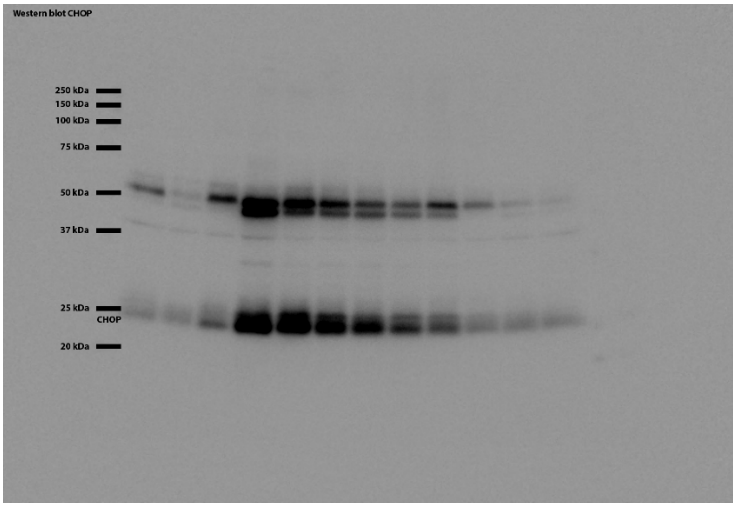

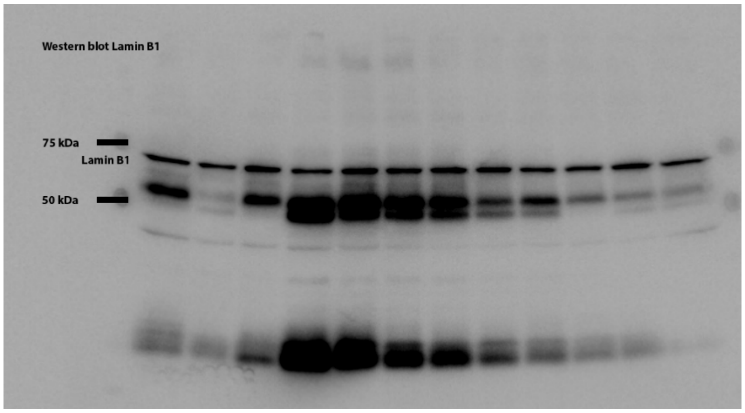

CHOP and the associated loading control Lamin B1 – II:

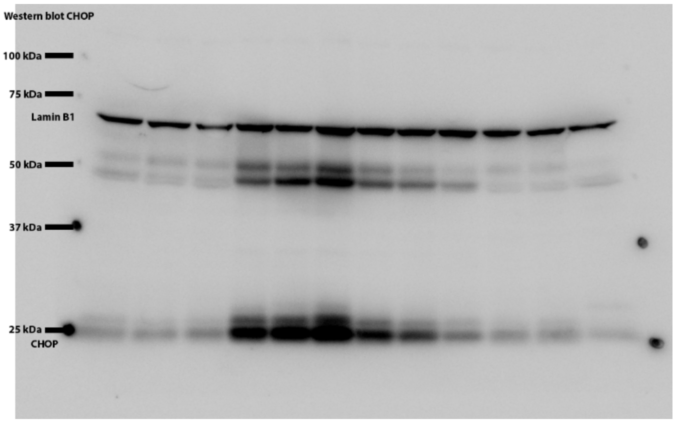

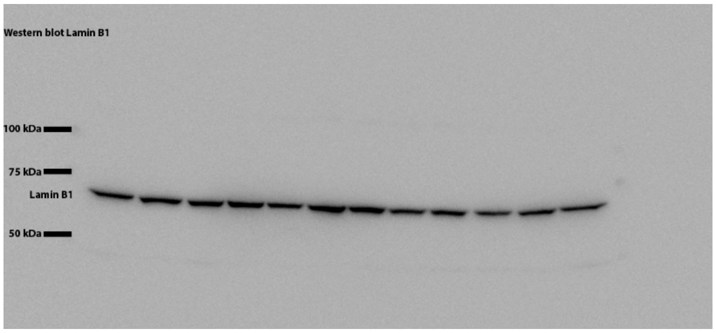

CHOP and the associated loading control Lamin B1 – III:

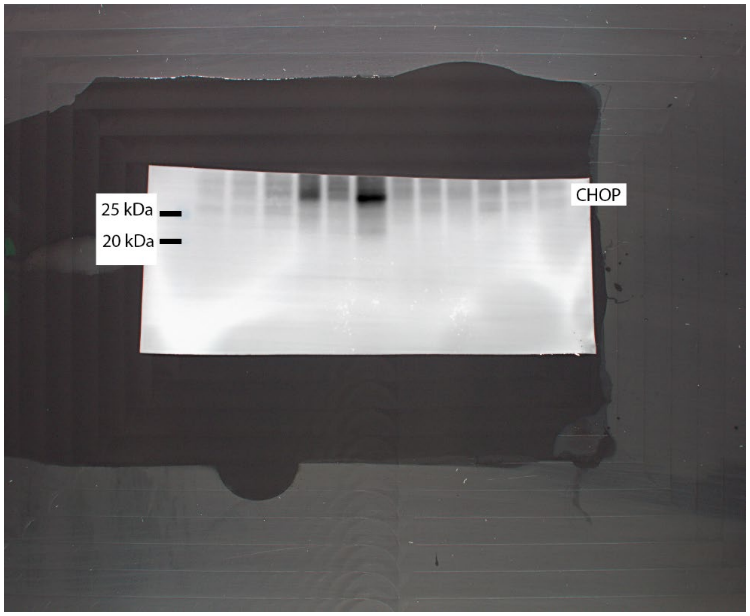

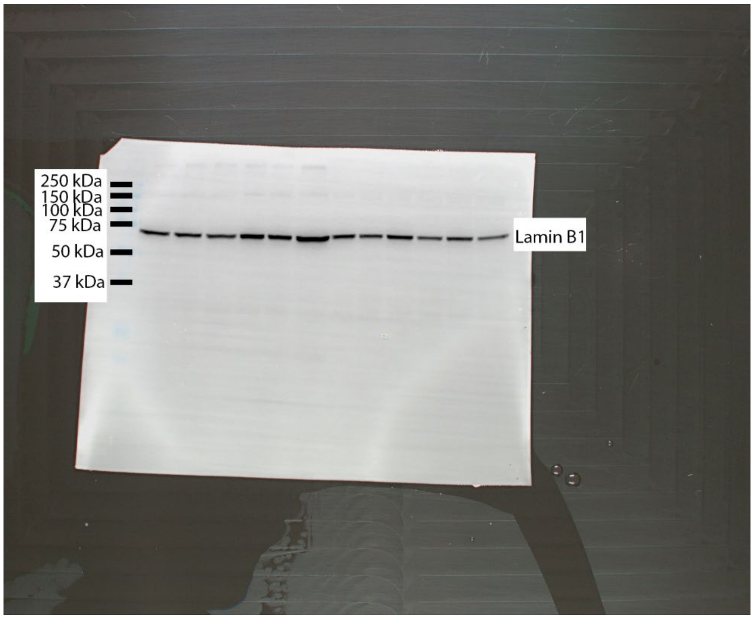

CHOP and the associated loading control Lamin B1 – IV:

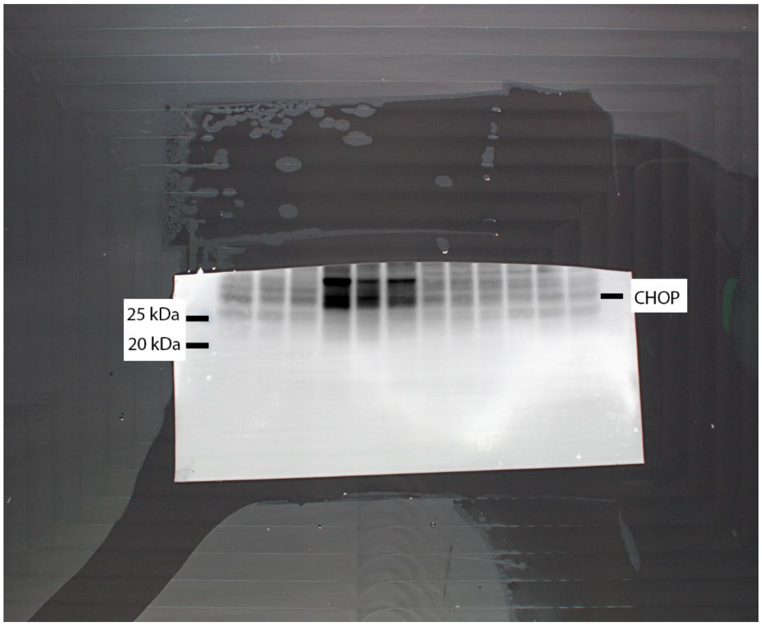

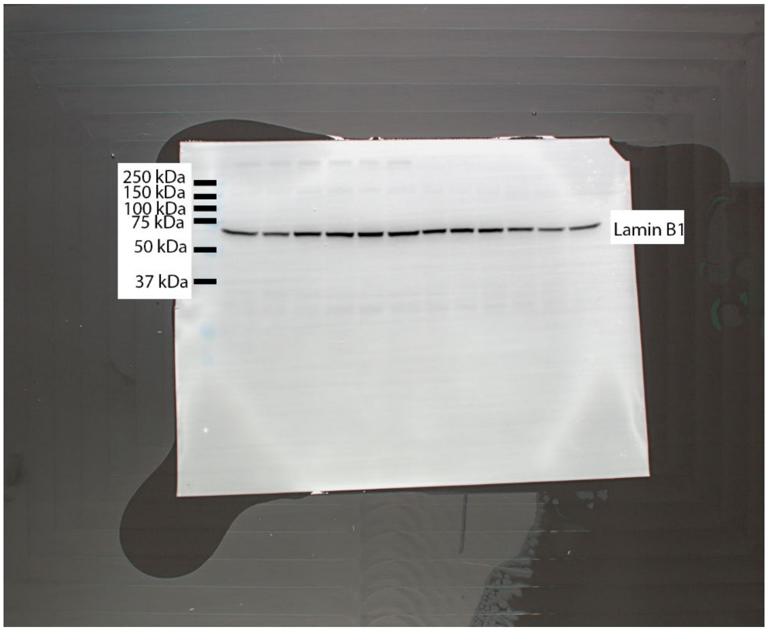

ERp72 and the associated loading control Lamin B1 – I:

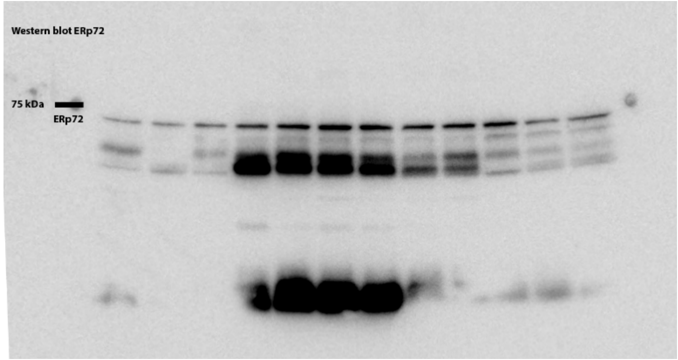

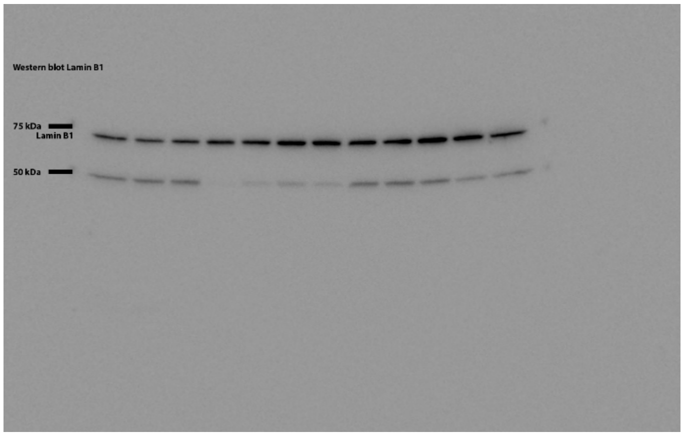

ERp72 and the associated loading control Lamin B1 – II:

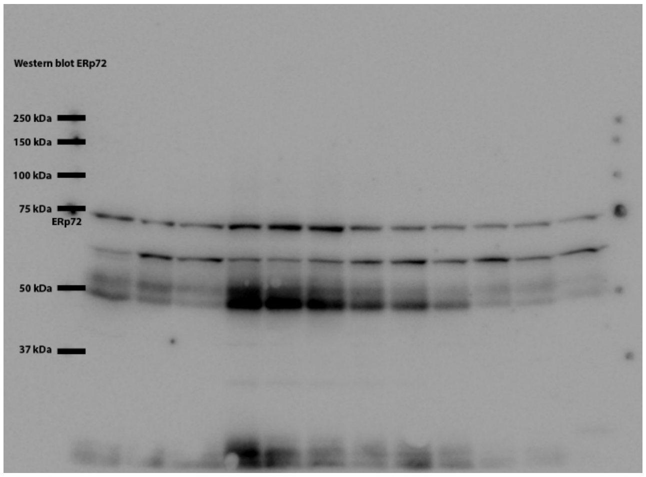

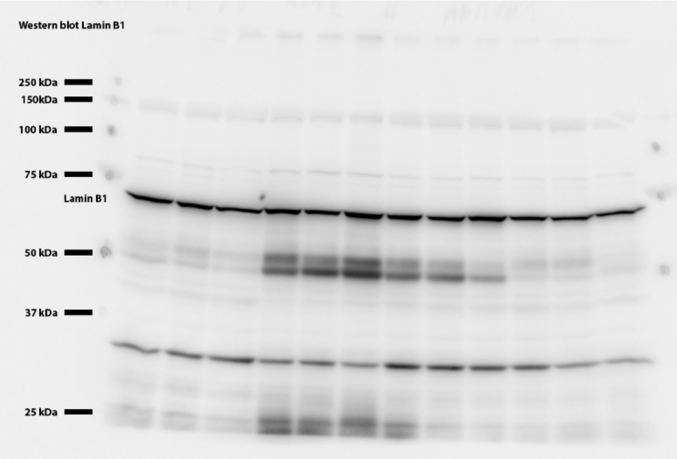

IRE1α and the associated loading control Lamin B1 – I:

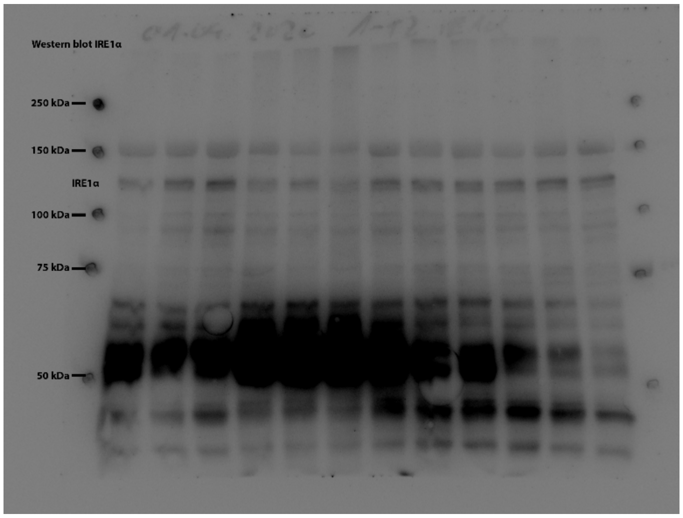

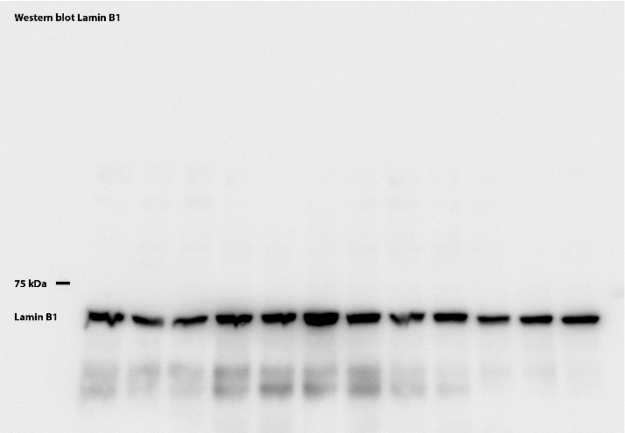

IRE1α and the associated loading control Lamin B1 – II:

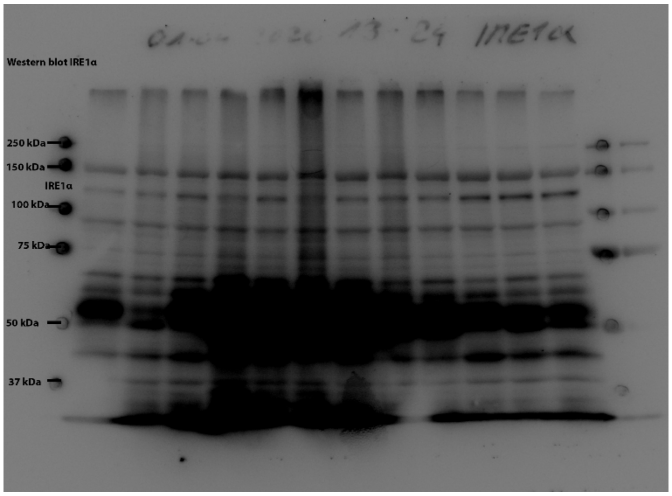

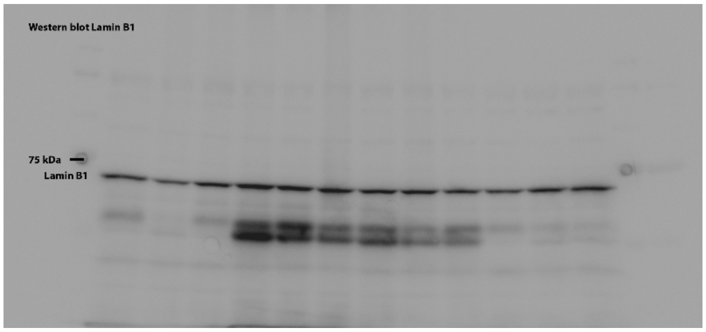

IRE1α and the associated loading control Lamin B1 – III:

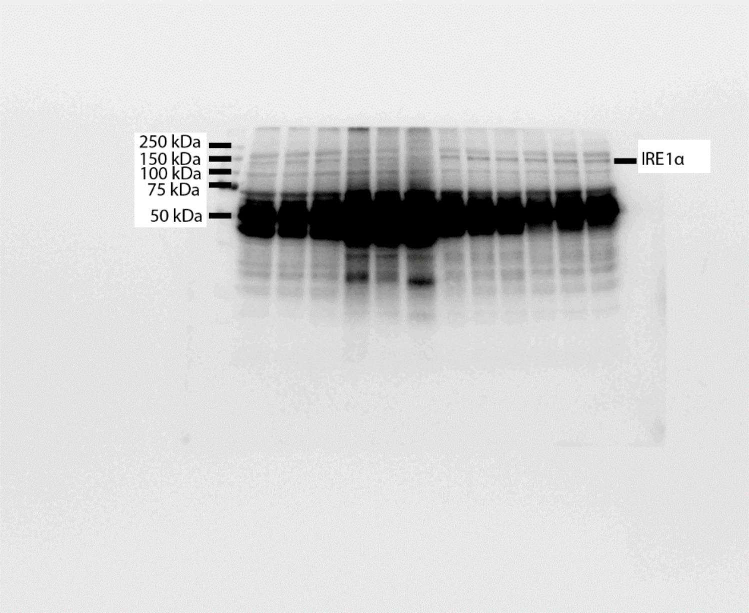

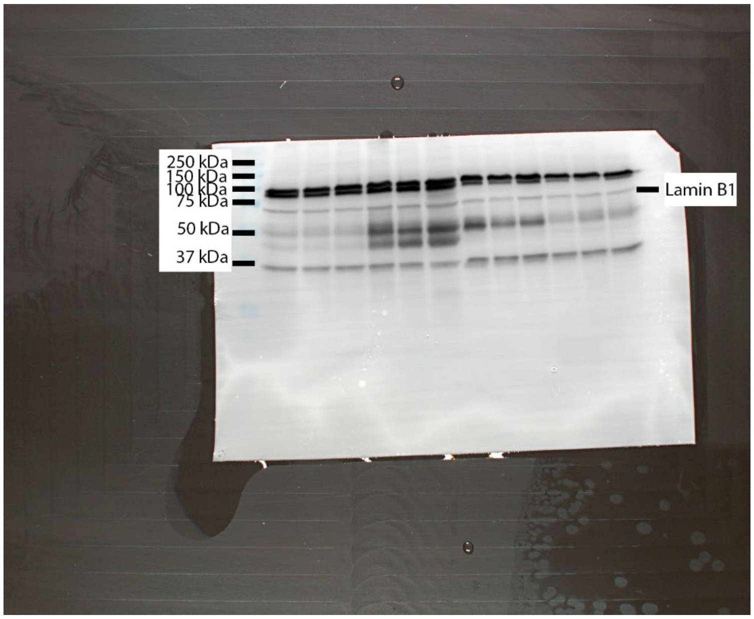

IRE1α and the associated loading control Lamin B1 – IV:

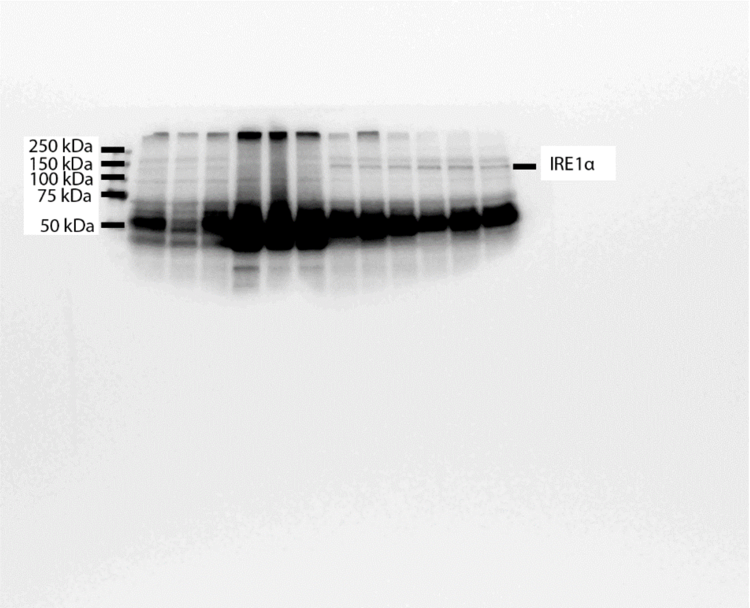

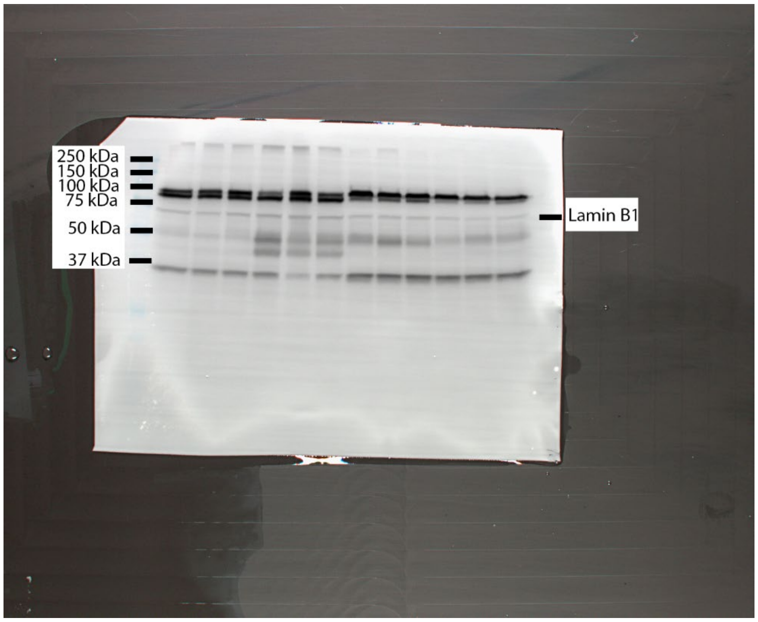

p-IRE1α and the associated loading control Lamin B1 – I:

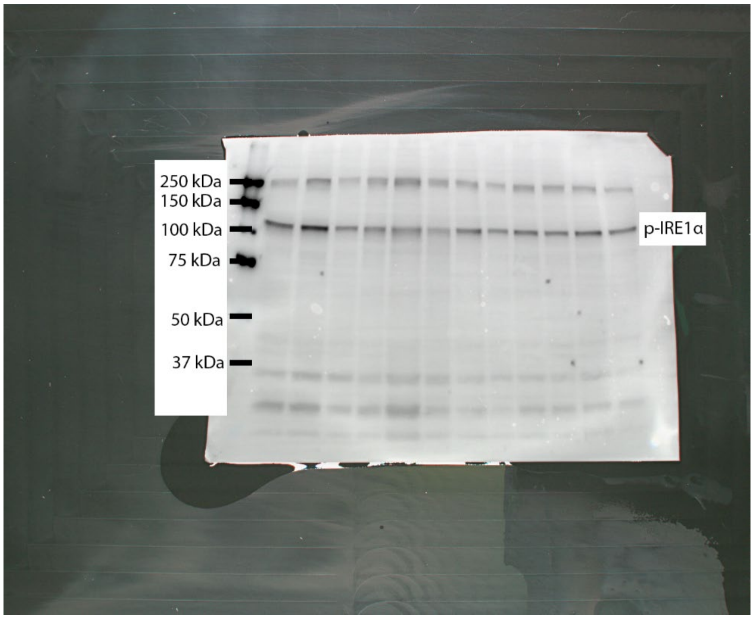

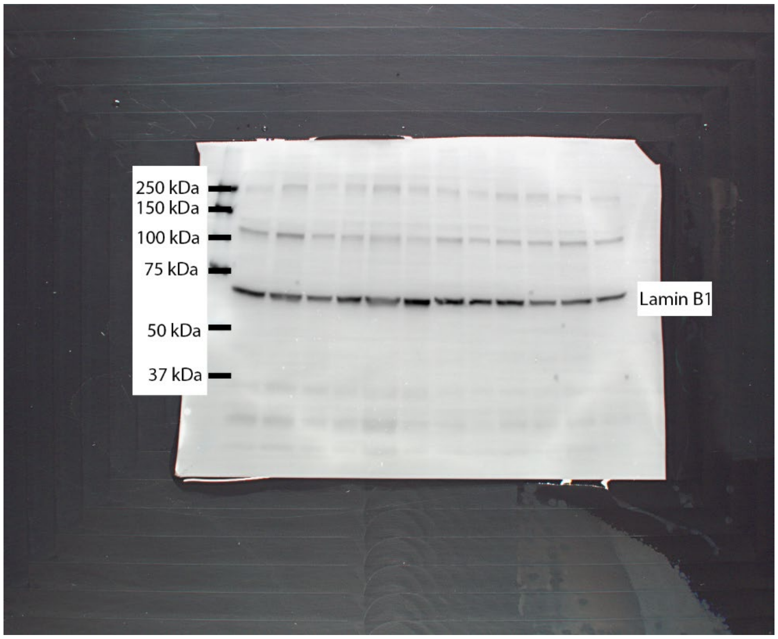

p-IRE1α and the associated loading control Lamin B1 – II:

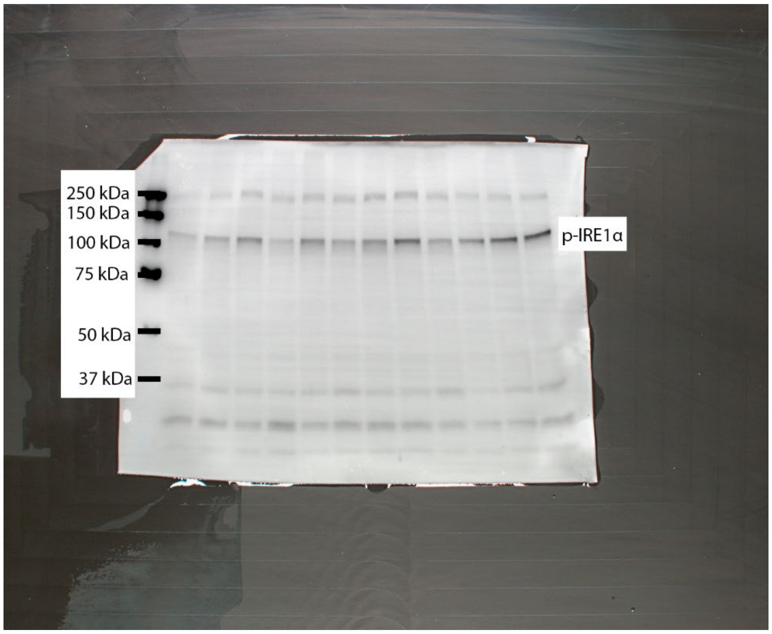

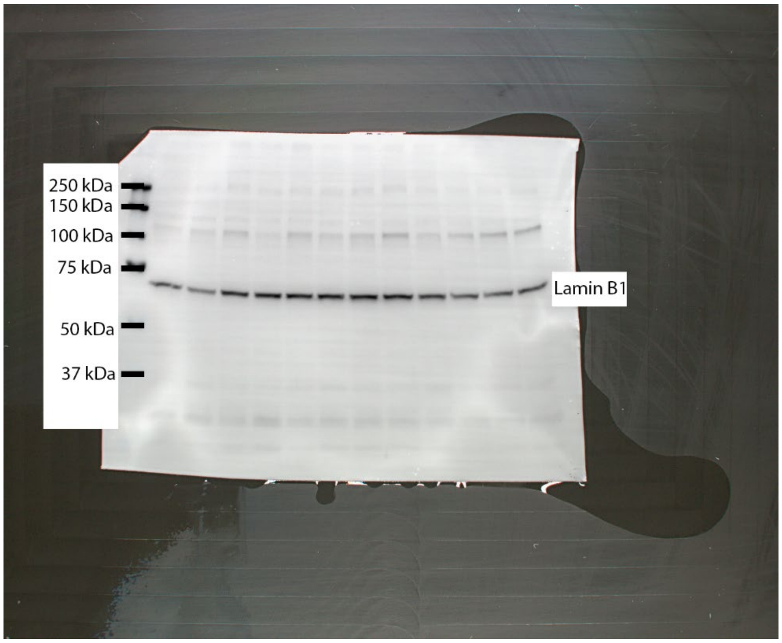

Calnexin and the associated loading control Lamin B1 – I:

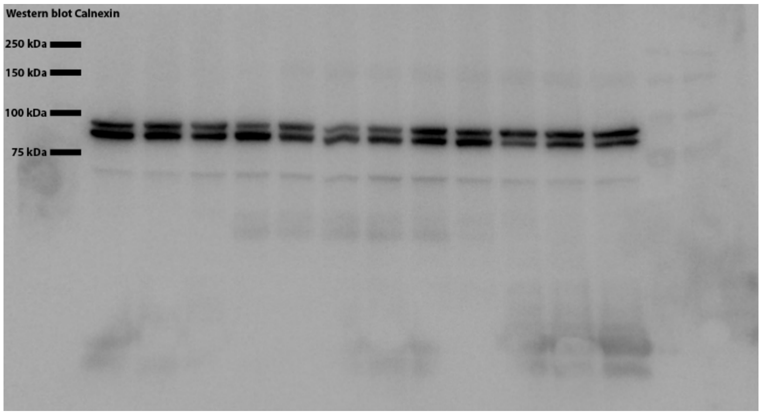

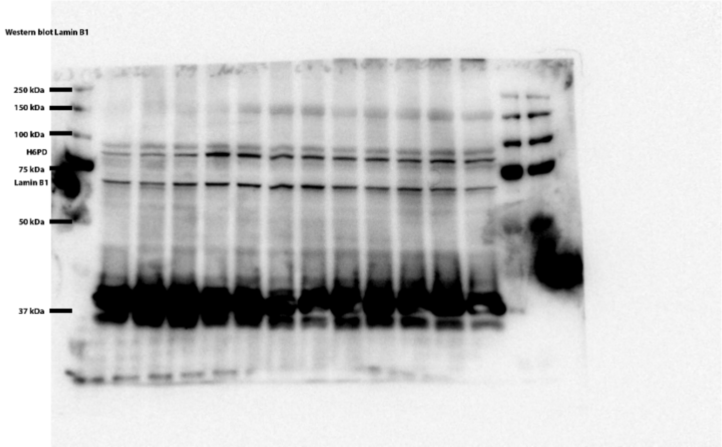

Calnexin and the associated loading control Lamin B1 – II:

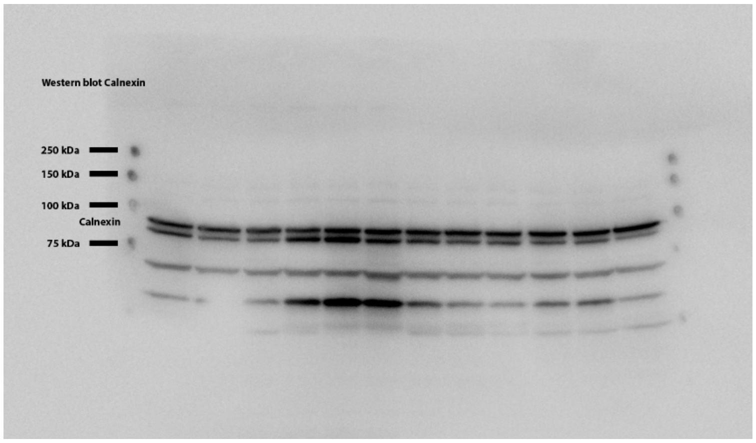

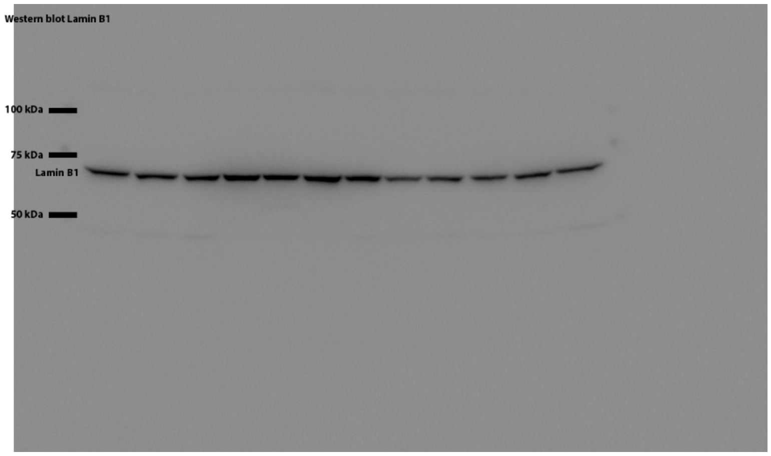

Calreticulin and the associated loading control Lamin B1 – I:

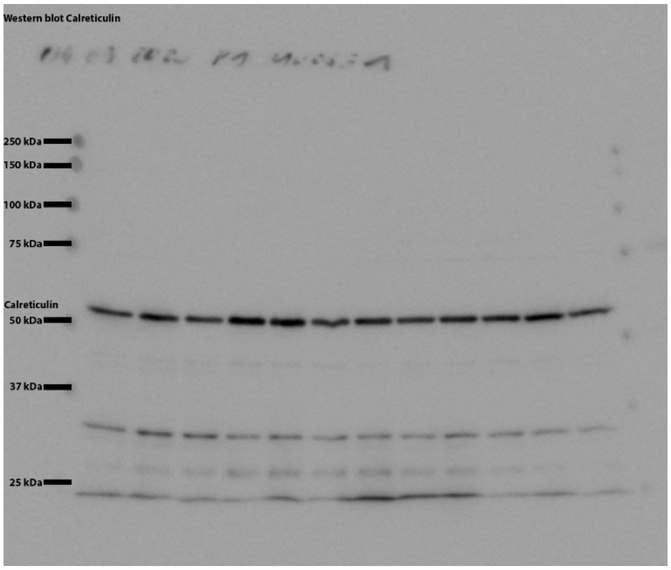

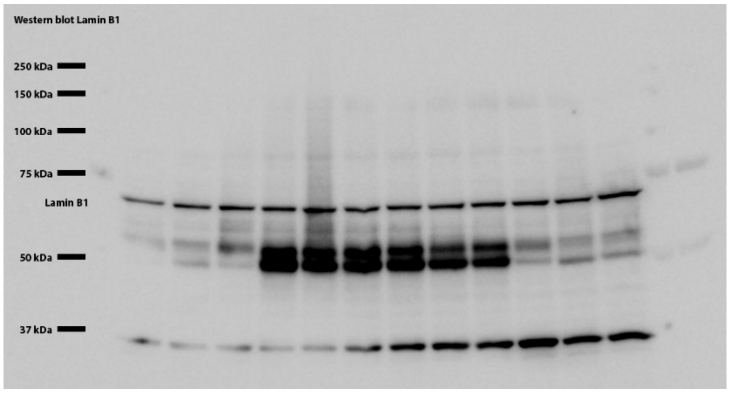

Calreticulin and the associated loading control Lamin B1 – II:

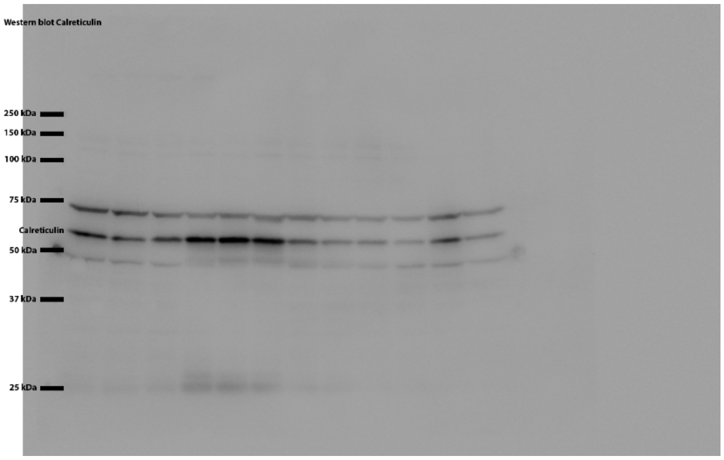

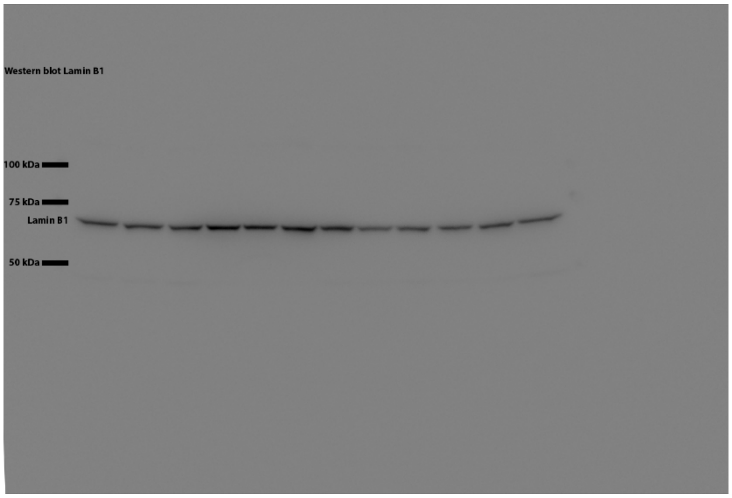

H6PD and the associated loading control Lamin B1 – I:

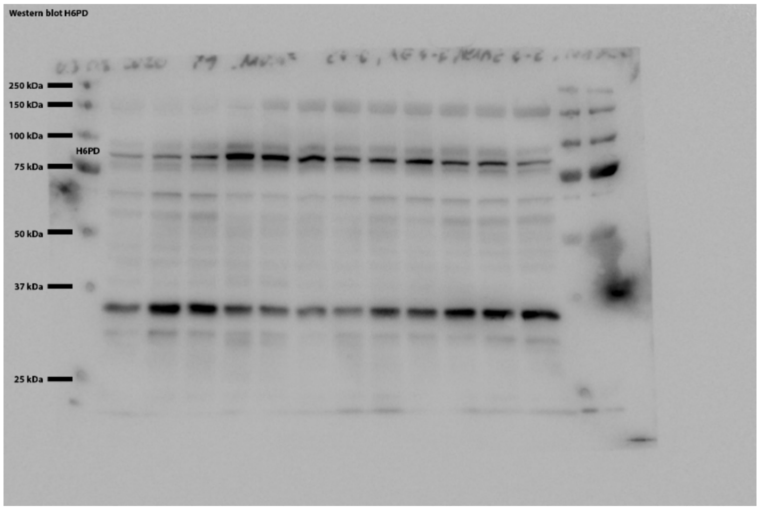

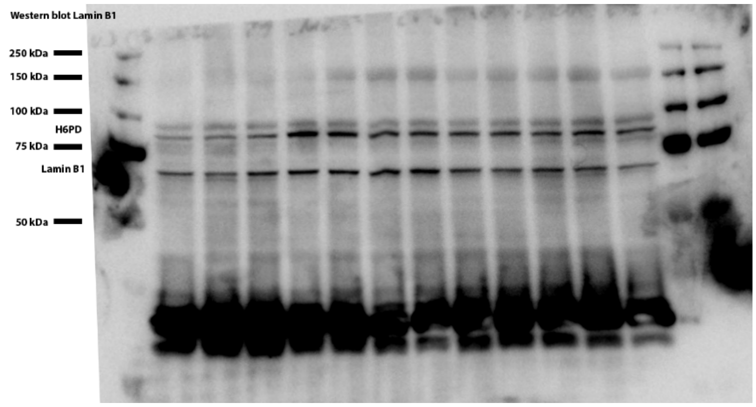

H6PD and the associated loading control Lamin B1 – II:

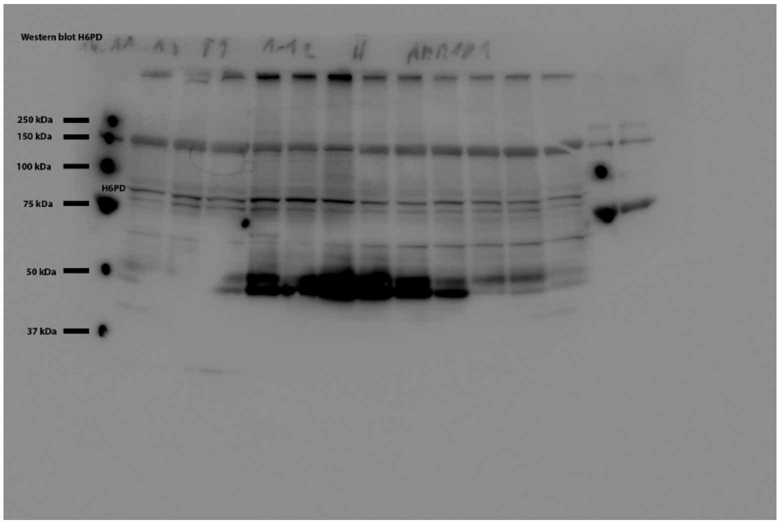

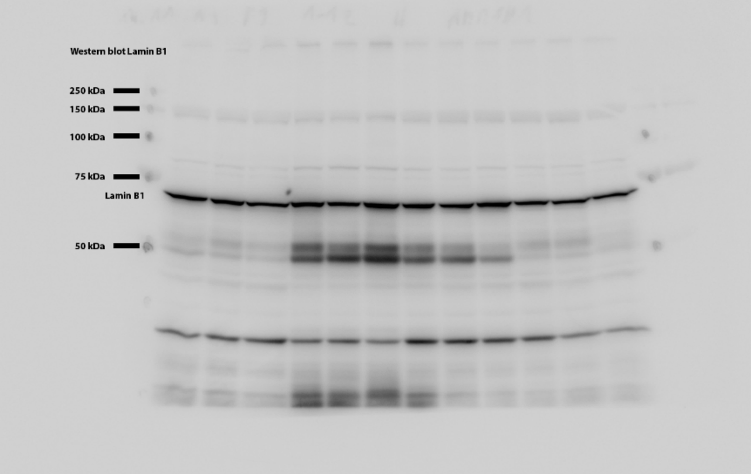

Supplement: S1 File — (PDF) [file pntd.0009192.s008.pdf]
